# Calcium Orthophosphates in Nature, Biology and Medicine

**DOI:** 10.3390/ma2020399

**Published:** 2009-04-20

**Authors:** Sergey V. Dorozhkin

**Affiliations:** Kudrinskaja sq. 1-155, Moscow 123242, Russia; E-Mail: sedorozhkin@yandex.ru; Tel. +7-499-255-4460

**Keywords:** Calcium orthophosphates, antlers, biological apatite, biomaterials, bioceramics, biomimetics, biomineralization, bone grafts, bones, calcified tissues, fluorapatite, hydroxyapatite, materials chemistry, pathological calcification, teeth, tissue engineering

## Abstract

The present overview is intended to point the readers’ attention to the important subject of calcium orthophosphates. These materials are of the special significance because they represent the inorganic part of major normal (bones, teeth and dear antlers) and pathological (*i.e.* those appearing due to various diseases) calcified tissues of mammals. Due to a great chemical similarity with the biological calcified tissues, many calcium orthophosphates possess remarkable biocompatibility and bioactivity. Materials scientists use this property extensively to construct artificial bone grafts that are either entirely made of or only surface-coated with the biologically relevant calcium ortho-phosphates. For example, self-setting hydraulic cements made of calcium orthophosphates are helpful in bone repair, while titanium substitutes covered by a surface layer of calcium orthophosphates are used for hip joint endoprostheses and as tooth substitutes. Porous scaffolds made of calcium orthophosphates are very promising tools for tissue engineering applications. In addition, technical grade calcium orthophosphates are very popular mineral fertilizers. Thus ere calcium orthophosphates are of great significance for humankind and, in this paper, an overview on the current knowledge on this subject is provided.

## 1. Introduction

Calcium orthophosphates are chemical compounds of special interest in many interdisciplinary fields of science, including geology, chemistry, biology and medicine. According to the literature, the initial attempts to establish their chemical composition were performed by J. Berzelius in the middle of the 19^th^ century [1]. However, the first systematic studies were performed by F. K. Cameron and co-workers [2,3,4,5] and H. Bassett [6,7,8,9] at the beginning of the 20^th^ century. Both researchers already worked with individual chemical compounds of various calcium orthophosphates, which had been called apatites [10] until then [11].

By definition, all calcium orthophosphates consist of three major chemical elements: calcium (oxidation state +2), phosphorus (oxidation state +5) and oxygen (reduction state – 2), as a part of orthophosphate anions. These three chemical elements are present in abundance on the surface of our planet: oxygen is the most widespread chemical element of the Earth's surface (~ 47 mass %), calcium occupies the fifth place (~ 3.3 – 3.4 mass %) and phosphorus (~ 0.08 – 0.12 mass %) is among the first twenty chemical elements most widespread on our planet [12]. In addition, the chemical composition of many calcium orthophosphates includes hydrogen, either as part of an acidic orthophosphate anion (for example, HPO_4_^2-^ or H_2_PO_4_^-^), hydroxide (for example, Ca_10_(PO_4_)_6_(OH)_2_) and/or incorporated water (for example, CaHPO_4_·2H_2_O). Diverse combinations of CaO and P_2_O_5_ (both in the presence of water and without it) provide a large variety of calcium phosphates, which are distinguished by the type of the phosphate anion: ortho- (PO_4_^3-^), meta- (PO_3_^-^), pyro- (P_2_O_7_^4-^) and poly- ((PO_3_)_n_^n-^). In the case of multi-charged anions (orthophosphates and pyrophosphates), calcium phosphates are also differentiated by the number of hydrogen ions attached to the anion. Examples include mono- (Ca(H_2_PO_4_)_2_), di- (CaHPO_4_), tri- (Ca_3_(PO_4_)_2_) and tetra- (Ca_2_P_2_O_7_) calcium phosphates [13,14,15]. However, only the various calcium orthophosphates will be reviewed in this paper.

The atomic arrangement of calcium orthophosphates is built up around a network of orthophosphate (PO_4_) groups, which provides stability to the entire structure. The majority of calcium orthophosphates are sparingly soluble in water and insoluble in alkaline solutions, but all of them are easily soluble in acids. All chemically pure calcium orthophosphates are white colored crystals of moderate hardness, whereas natural calcium orthophosphates minerals are always colored due to the presence of different impurities, the most widespread of which are ions of Fe, Mn and rare earth elements [16,17]. Biologically formed calcium orthophosphates are the major component of all mammalian calcified tissues [8], while the natural ones are the major raw materials used to produce phosphorus-containing fertilizers [19,20,21,22].

## 2. Geological and Biological Ooccurrence of Calcium Orthophosphates

Calcium orthophosphates are abundant in both nature and living organisms. Geologically, natural calcium orthophosphates are found in different regions, mostly as deposits of apatites (whch belong to the igneous rocks), mainly as natural fluorapatite (FA, chemical formula Ca_10_(PO_4_)_6_F_2_) or phosphorites (a sedimentary rock) [20,23]. Some types of sedimentary rocks can be formed by weathering of igneous rocks into smaller particles. Other types of sedimentary rocks can be composed of minerals precipitated from the dissolution products of igneous rocks or minerals produced by biomineralization. Thus, due to a sedimentary origin, both a general appearance and a chemical composition of natural phosphorites vary a lot. It is a common practice to consider francolite (or carbonate-hydroxyfluorapatite regarded as its synonym) as the basic phosphorite mineral [23,24,25,26,27,28]. A cryptocrystalline (almost amorphous) variety of francolite (partly of a biological origin) is called collophane (synonyms: collophanit, collophanita, collophanite, grodnolite, kollophan) [29,30]. It occurs in natural phosphorites predominantly as fossil bones and phosphatized microbial pseudomorphs: phosphatic crusts of chasmolithic biofilms (or microstromatolites) and globular clusters with intra-particular porosities [31,32]. Natural phosphorites (therefore, francolite and collophane as well) occur in various forms, such as nodules, crystals, or masses. Occasionally, other types of natural calcium orthophosphates are found as minerals, for example clinohydroxylapatite [33] and staffelite (synonyms: staffelit, staffelita) belonging to carbonate-rich fluorapatites (chemical formula: Ca_5_[(F,O)(PO_4_,CO_3_)_3_]) [34], as well as CaHPO_4_·2H_2_O [35]. Furthermore, calcium orthophosphates have been found in meteorite stones [36]. The world deposits of natural calcium orthophosphates are estimated to exceed 150 billion tons, of which approximately 85 % are phosphorites and the remaining ~ 15 % are apatites [23].

**Figure 1:**
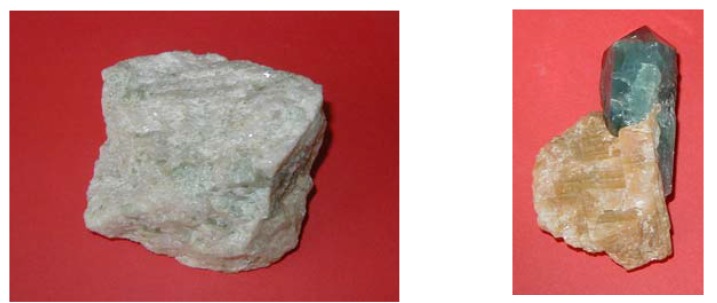
Polycrystalline **(left)** and single-crystalline **(right)** FA of a geological origin. The single crystal has a grey-green color due to incorporated ions of transition metals [16,17].

Natural calcium orthophosphates occur in most geological environments, usually as accessory minerals (< 5 %). Concentrations sufficient for economic use (> 15 %) are also available. The largest world deposits of natural apatites are located in Russia (the Khibiny and Kovdor massifs, Kola peninsula [37]), Brazil and Zambia, while the largest world deposits of natural phosphorites are located in Morocco, Russia, Kazakhstan, USA (Florida, Tennessee), China and Australia, as well as in the oceans [19,20,21,22,23]. Most of natural calcium orthophosphates occur as small polycrystalline structures (spherulitic clusters). Larger crystals are rare [24]. They usually have the crystal structure of apatites (hexagonal system, space group *P*6_3_/m). Giant crystals including “a solid but irregular mass of green crystalline apatite, 15 feet long and 9 feet wide” and a single euhedral crystal from the Aetna mine measuring 2.1 × 1.2 m with an estimated weight of six tons have been found [25,26]. None of them is a pure compound; they always contain admixtures of other elements. For example, ions of calcium might be partially replaced by Sr, Ba, Mg, Mn, K, Na, Fe; ions of orthophosphate may be partly replaced by AsO_4_^3-^, CO_3_^2-^ and VO_4_^3-^ [38]; ions of hydroxide, chloride, bromide, carbonate and oxide may to a certain extent substitute fluoride in the crystal lattice of natural apatites [27]. In principle, the crystal structure of apatites can incorporate half the periodic table in its atomic arrangement. Ease of atomic substitution for apatite leaves this mineral open to a wide array of compositions. This might be related to the fact that the apatite structure type displays porous properties [39]. The substitutions in apatites are usually in trace concentrations but large concentrations and even complete solid solutions exist for certain substituents (*e.g.*, F^-^ and OH^-^). To make things even more complicated, some ions in the crystal structure may be missing, leaving the crystallographic defects, which leads to formation of non-stoichiometric compounds. Figure 1 shows examples of polycrystalline and single-crystalline samples of natural FA.

The manufacture of elementary phosphorus (white and red) [40], phosphoric acids, various phosphorus-containing chemicals and, especially, agricultural fertilizers (namely, superphosphate [41,42] and ammonium orthophosphates [43]) are the major industrial applications of natural calcium orthophosphates. This consumes up to 85% of the world production. The total capacity of industrial plants in the world exceeds 25 million tons (as P_2_O_5_) of phosphate fertilizers per year with an annual increase of 2 – 3 % [20].

In biological systems, many organisms, ranging from bacteria and isolated cells to invertebrates and vertebrates, synthesize calcium orthophosphates [44]. Formation of calcium orthophosphates in primitive organisms is believed to enable the storage and regulation of essential elements such as calcium, phosphorus and, possibly, magnesium. The morphology of precipitates in these organisms (small intracellular nodules of amorphous calcium phosphates often located in mitochondria) complies with the necessities for rapid mobilization and intracellular control of the concentration of these elements [45]. In vertebrates calcium orthophosphates occur as the principal inorganic constituent of normal (bones, teeth, fish enameloid, deer antlers and some species of shells) and pathological (dental and urinary calculus and stones, atherosclerotic lesions, *etc*.) calcifications [13,46,47,48,49,50,51]. Except for small portions of the inner ear, all hard tissue of the human body is formed of calcium orthophosphates. Structurally, they occur mainly in the form of poorly crystallized non-stoichiometric Na-, Mg- and carbonate-containing hydroxyapatite (often called biological apatite [52] or dahllite [56]). The main constituents of human bones are calcium orthophosphates (~ 60 – 70 wt%), collagen [60] (~ 20 – 30 wt%) and water (up to 10 wt%) [50,53,54,55,61,62,63]. Detailed information on the chemical composition of the most important human normal calcified tissues is compiled in Table 1. One should note that the values mentioned in Table 1 are approximate; the main constituents can vary by a percent or more [64].

## 3. The Members of the Calcium Orthophosphate Family

In the ternary system Ca(OH)_2_ – H_3_PO_4_ – H_2_O (or CaO – P_2_O_5_ – H_2_O) [65,66,67] there are eleven [68] known non-ion-substituted calcium orthophosphates with the Ca/P molar ratio within 0.5 and 2.0 (Table 2). Table 3 lists their crystallographic data [14,73,74,75]. The most important parameters are the molar Ca/P ratio, basicity/acidity and solubility. These parameters strongly correlate with the solution pH. The lower the Ca/P molar ratio is, the more acidic and water-soluble the calcium orthophosphate is [13,14,15]. One can see that the solubility ranges from high values for acidic compounds, such as MCPM, to very low values for basic compounds, such as apatites, which allow calcium orthophosphates to be dissolved, transported from one place to another and precipitated, when necessary. Crystallization, dissolution and phase transformation processes of different calcium orthophosphates under various experimental conditions have been reviewed recently [76].

**Table 1:** Comparative composition and structural parameters of inorganic phases of adult human calcified tissues. Due to the considerable variation found in biological samples, typical values are given in these cases [13,63].

Composition, wt.%	Enamel	Dentin	Cementum	Bone	HA
Calcium^[a]^	36.5	35.1	^[c]^	34.8	39.6
Phosphorus (as P)^[a]^	17.7	16.9	^[c]^	15.2	18.5
Ca/P (molar ratio)^[a]^	1.63	1.61	^[c]^	1.71	1.67
Sodium^[a]^	0.5	0.6	^[c]^	0.9	-
Magnesium^[a]^	0.44	1.23	^[c]^	0.72	-
Potassium^[a]^	0.08	0.05	^[c]^	0.03	-
Carbonate (as CO_3_^2-^)^[b]^	3.5	5.6	^[c]^	7.4	-
Fluoride^[a]^	0.01	0.06	^[c]^	0.03	-
Chloride^[a]^	0.30	0.01	^[c]^	0.13	-
Pyrophosphate (as P_2_O_7_^4-^)^[b]^	0.022	0.10	^[c]^	0.07	-
Total inorganic^[b]^	97	70	60	65	100
Total organic^[b]^	1.5	20	25	25	-
Water^[b]^	1.5	10	15	10	-
**Crystallographic properties: Lattice parameters ( ± 0.003 Å)**					
*a*-axis, Å	9.441	9.421	^[c]^	9.41	9.430
*c*-axis, Å	6.880	6.887	^[c]^	6.89	6.891
Crystallinity index, (HA = 100)	70 – 75	33 – 37	^[c]^	33 – 37	100
Typical crystal sizes (nm) [311, 362, 364]	10^5^×50×50	35×25×4	^[c]^	50×25×4	200 – 600
Ignition products (800 ºC)	β-TCP + HA	β-TCP+ HA	β-TCP+ HA	HA + CaO	HA
Elastic modulus (GPa)	80	15	^[c]^	0.34 – 13.8	10
Tensile strength (MPa)	10	100	^[c]^	150	100

^[a]^ Ashed samples.

^[b]^ Unashed samples.

^[c]^ Numerical values were not found in the literature but they should be similar to those for dentin.

**Figure 2:**
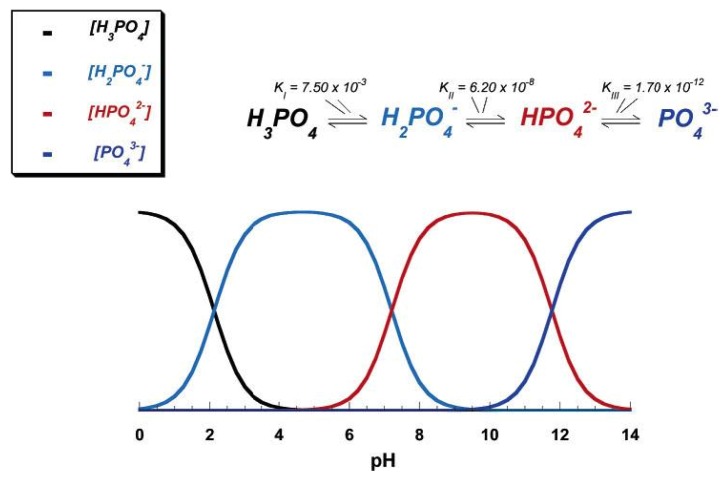
pH variation of ionic concentrations in triprotic equilibrium for phosphoric acid solutions. Reprinted from Ref. [77] with permission.

Due to the triprotic equilibrium that exists within orthophosphate-containing solutions, variations in pH alter the relative concentrations of the four polymorphs of orthophosphoric acid (Figure 2) and thus both the chemical composition and the amount of the calcium orthophosphates that forms by direct precipitation [77]. The solubility isotherms of different calcium orthophosphates are available in literature [66,67,78,79]. However, very recently, the classic solubility data of calcium orthophosphates [66,67,78,79] were mentioned to be inappropriate [80]. According to the authors, all previous solubility calculations were based on simplifications, which are only crudely approximate. The problem lies in incongruent dissolution, leading to phase transformations and lack of the detailed solution equilibria. Using an absolute solid-titration approach, the true solubility isotherm of HA was found to lie substantially lower than previously reported. In addition, contrary to a wide belief, DCPD appeared not to be the most stable phase below pH ~ 4.2, where CDHA was less soluble [80]. A brief description of all calcium orthophosphates is given below.

**Table 2:** Existing calcium orthophosphates and their major properties [78,79].

Ca/P ionic ratio	Compound	Chemical formula	Solubility at 25 ºC, –log(K_s_)	Solubility at 25 ºC, g/L	pH stability range in aqueous solutions at 25°C
0.5	Monocalcium phosphate monohydrate (MCPM)	Ca(H_2_PO_4_)_2_·H_2_O	1.14	~ 18	0.0 – 2.0
0.5	Monocalcium phosphate anhydrous (MCPA)	Ca(H_2_PO_4_)_2_	1.14	~ 17	^[c]^
1.0	Dicalcium phosphate dihydrate (DCPD), mineral brushite	CaHPO_4_·2H_2_O	6.59	~ 0.088	2.0 – 6.0
1.0	Dicalcium phosphate anhydrous (DCPA), mineral monetite	CaHPO_4_	6.90	~ 0.048	^[c]^
1.33	Octacalcium phosphate (OCP)	Ca_8_(HPO_4_)_2_(PO_4_)_4_·5H_2_O	96.6	~ 0.0081	5.5 – 7.0
1.5	α-Tricalcium phosphate (α-TCP)	α-Ca_3_(PO_4_)_2_	25.5	~ 0.0025	^[a]^
1.5	β-Tricalcium phosphate (β-TCP)	β-Ca_3_(PO_4_)_2_	28.9	~ 0.0005	^[a]^
1.2 – 2.2	Amorphous calcium phosphate (ACP)	Ca_x_H_y_(PO_4_)_z_·nH_2_O, n = 3 – 4.5; 15 – 20% H_2_O	^[b]^	^[b]^	~ 5 – 12 ^[d]^
1.5 – 1.67	Calcium-deficient hydroxyapatite (CDHA)^[e]^	Ca_10- *x*_(HPO_4_)_*x*_(PO_4_)_6-*x*_(OH)_2-*x*_^[f]^ (0<*x*<1)	~ 85.1	~ 0.0094	6.5 – 9.5
1.67	Hydroxyapatite (HA)	Ca_10_(PO_4_)_6_(OH)_2_	116.8	~ 0.0003	9.5 – 12
1.67	Fluorapatite (FA)	Ca_10_(PO_4_)_6_F_2_	120.0	~ 0.0002	7 – 12
2.0	Tetracalcium phosphate (TTCP), mineral hilgenstockite	Ca_4_(PO_4_)_2_O	38 – 44	~ 0.0007	^[a]^

^[a]^ These compounds cannot be precipitated from aqueous solutions.

^[b]^ Cannot be measured precisely. However, the following values were found: 25.7 ± 0.1 (pH = 7.40), 29.9 ± 0.1 (pH = 6.00), 32.7 ± 0.1 (pH = 5.28).

^[c]^ Stable at temperatures above 100°C.

^[d]^ Always metastable.

^[e]^ Occasionally, CDHA is named as precipitated HA.

^[f]^ In the case *x* = 1 (the boundary condition with Ca/P = 1.5), the chemical formula of CDHA looks as follows: Ca_9_(HPO_4_)(PO_4_)_5_(OH).

**Table 3:** Crystallographic data of calcium orthophosphates [14,73,74,75].

Compound	Space group	Unit cell parameters	Z^[a]^	Density, g cm^-3^
MCPM	triclinic *P* triclinic P $\bar{1}$	*a* = 5.6261(5), *b* = 11.889(2), *c* = 6.4731(8) Å,	2	2.23
		*α* = 98.633(6)º, *β* = 118.262(6)º, *γ* = 83.344(6)º		
MCPA	triclinic *P* triclinic P $\bar{1}$	*a* = 7.5577(5), *b* = 8.2531(6), *c* = 5.5504(3) Å,	2	2.58
		*α* = 109.87(1)º, *β* = 93.68(1)º, *γ* = 109.15(1)º		
DCPD	monoclinic *I*a	*a* = 5.812(2), *b* = 15.180(3), *c* = 6.239(2) Å, *β* = 116.42(3)º	4	2.32
DCPA	triclinic *P* triclinic P $\bar{1}$	*a* = 6.910(1), *b* = 6.627(2), *c* = 6.998(2) Å,	4	2.89
		*α* = 96.34(2)º, *β* = 103.82(2)º, *γ* = 88.33(2)º		
OCP	triclinic *P* triclinic P $\bar{1}$	*a* = 19.692(4), *b* = 9.523(2), *c* = 6.835(2) Å, *α* = 90.15(2)º, *β* = 92.54(2)º, *γ* = 108.65(1)º	1	2.61
α-TCP	monoclinic *P*2_1_/a	*a* = 12.887(2), *b* = 27.280(4), *c* = 15.219(2) Å, *β* = 126.20(1)º	24	2.86
β-TCP	rhombohedral *R*3cH	*a* = *b* = 10.4183(5), *c* = 37.3464(23) Å, *γ* = 120°	21^[b]^	3.08
HA	monoclinic *P*2_1_/b	*a* = 9.84214(8), *b* = 2*a*, *c* = 6.8814(7) Å, *γ* = 120° (monoclinic);	4	3.16
	or hexagonal *P*6_3_/m	*a* = *b* = 9.4302(5), *c* = 6.8911(2) Å, *γ* = 120º (hexagonal)	2	
FA	hexagonal *P*6_3_/m	*a* = *b* = 9.367, *c* = 6.884 Å, *γ* = 120º	2	3.20
TTCP	monoclinic *P*2_1_	*a* = 7.023(1), *b* = 11.986(4), *c* = 9.473(2) Å, *β* = 90.90(1)º	4	3.05

^[a]^ Number of formula units per unit cell.

^[b]^ Per the hexagonal unit cell.

### 3.1. MCPM

MCPM (monocalcium phosphate monohydrate, Ca(H_2_PO_4_)_2_·H_2_O; the chemically correct name is calcium dihydrogen phosphate monohydrate) is both the most acidic and water-soluble compound. It precipitates from highly acidic solutions that are normally used in industry of phosphorus-containing fertilizer production (“triple superphosphate”) [20]. At temperatures above 100 ºC, it releases a molecule of water and transforms into MCPA. Due to high acidity and solubility, MCPM is never found in biological calcifications. Moreover, pure MCPM is not biocompatible [81] with bone [83]. However, MCPM is used in medicine as a component of several self-hardening calcium orthophosphate cements [84,85,86,87]. In addition, MCPM is used as a nutrient, acidulant and mineral supplement for dry baking powders, food, feed and some beverages [88,89]. Coupled with NaHCO_3_, MCPM is used as a leavening agent for both dry baking powders and bakery dough. MCPM might be added to salt-curing preserves, pickled and marinated foods. According to the European Classification of Food Additives, MCPM is marked as additive E341 . Occasionally, MCPM is added to toothpastes. In addition, MCPM might be added to ceramics and glasses, while agriculture is the main consumer of a technical grade MCPM, where it is used as a fertilizer [20,88].

### 3.2. MCPA

MCPA (monocalcium phosphate anhydrous, Ca(H_2_PO_4_)_2_; the chemically correct name is calcium dihydrogen phosphate anhydrous) is the anhydrous form of MCPM. It crystallizes under the same conditions as MCPM, but at temperatures above 100 ºC (*e.g.*, from highly concentrated mother liquors during fertilizer production). Like MCPM, MCPA never appears in calcified tissues and is not biocompartible due to its acidity. There is no current application of MCPA in medicine. Due to the similarity with MCPM, in many cases, MCPA might be used instead of MCPM [20,88]; however, its hydroscopic properties reduce its commercial applications.

### 3.3. DCPD

DCPD (dicalcium phosphate dihydrate, CaHPO_4_·2H_2_O; the chemically correct name is calcium hydrogen phosphate dihydrate; the mineral brushite [90]) can be easily crystallized from aqueous solutions at pH < 6.5. It transforms into DCPA at temperatures above 80 ºC. Briefly, DCPD crystals consist of CaPO_4_ chains arranged parallel to each other, while lattice water molecules are interlayered between them. Using surface X-ray diffraction, Arsic *et al*. determined the atomic structure of the {010} interface of DCPD with water [91]. Since DCPD contains water layers as part of its crystal structure, special ordering properties at the interface are expected. This interface consists of two water bilayers with different ordering properties. The first is highly ordered and can be considered as part of the DCPD crystal structure. Surprisingly, the second water bilayer exhibits no in-plane order but shows only layering in the perpendicular direction. It has been proposed that the low level of water ordering at the interface is correlated with the low solubility of DCPD in water [91]. Many additional data on DCPD, as well as a good picture of DCPD atomic structure are available in literature [92].

DCPD is of biological importance because it is often found in pathological calcifications (dental calculi, crystalluria, chondrocalcinosis and urinary stones) and some carious lesions [13,46,47,48]. It has been proposed as an intermediate in both bone mineralization and dissolution of enamel in acids (dental erosion) [13,46,47]. In medicine, DCPD is used in calcium orthophosphate cements [85,93,94,95,96] and as an intermediate for tooth remineralization. DCPD is added to toothpaste both for caries protection (in this case, it is coupled with F-containing compounds such as NaF and/or Na_2_PO_3_F) and as a gentle polishing agent [97,98,99,100,101]. Other applications include a flame retardant [102], a slow release fertilizer, glass production, as well as calcium supplement in food, feed and cereals [88]. The importance of DCPD as a constituent of infant’s food was discovered as early as in 1917 [103]. In food industry, it serves as a texturizer, bakery improver and water retention additive. In the diary industry, DCPD is used as a mineral supplement. If added to food products, DCPD should be identified as E341 according to the European Classification of Food Additives. In addition, plate-like crystals of DCPD might be used as a non-toxic, anticorrosive and passivating pigment for some basecoat paints.

### 3.4. DCPA

DCPA (dicalcium phosphate anhydrous, CaHPO_4_; the chemically correct name is calcium hydrogen phosphate anhydrous; the mineral monetite [104]) is the anhydrous form of DCPD. It is less soluble than DCPD due to the absence of water inclusions. Like DCPD, DCPA can be crystallized from aqueous solutions, but at 100 ºC. A calcium-deficient DCPA was prepared recently. It might be sintered at 300 ºC [105]. Unlike DCPD, DCPA occurs in neither normal nor pathological calcifications. It is used in calcium phosphate cements [95,106,107,108,109,110]. Other applcations include uses as a polishing agent, a source of calcium and phosphate in nutritional supplements (*e.g.,* in prepared breakfast cereals, enriched flour and noodle products), a tabletting aid and a toothpaste component [88]. In addition, it is used as a dough conditioner in the food industry.

### 3.5. OCP

OCP (octacalcium phosphate, Ca_8_(HPO_4_)_2_(PO_4_)_4_·5H_2_O; the chemically correct name is octacalcium bis(hydrogenphosphate) tetrakis(phosphate) pentahydrate [74]) is often found as an unstable transient intermediate during the precipitation of the thermodynamically more stable calcium orthophosphates (*e.g.*, CDHA) in aqueous solutions. Techniques for its preparation may be found elsewhere [111,112,113,114]. A partially hydrolyzed form of OCP with a Ca/P molar ratio of 1.37 can be prepared as well [115]. The full hydrolysis of OCP into CDHA occurs within 6 hours [116]. The triclinic structure of OCP displays apatitic layers (with atomic arrangements of calcium and orthophosphate ions similar to those of HA) separated by hydrated layers (with atomic arrangements of calcium and orthophosphate ions similar to those in DCPD) [13,14,15,117]. A similarity in crystal structure between OCP and HA is one reason that the epitaxial growth of these phases is observed. Morphologically, OCP crystallizes as {100} blades of triclinic pinacoidal symmetry, elongated along the *a*-axis and bordered by the forms {010}, {001} and {011}. It is generally assumed that, in solutions, the hydrated layer of the (100) face is the layer most likely exposed to solution. The water content of OCP crystals is about 1/5 that of DCPD and this is partly responsible for its lower solubility. 

OCP is of a great biological importance because it is one of the stable components of human dental and urinary calculi [118,119,120]. OCP was first proposed to participate as the initial phase in enamel mineral formation and bone formation through subsequent precipitation and stepwise hydrolysis of OCP by W. E. Brown [121,122,123]. It plays an important role in *in vivo* formation of apatitic biominerals. A “central OCP inclusion” (also known as “central dark line”) is seen by transmission electron microscopy in many biological apatites and in some synthetically precipitated HA [124,125,126,127]. Although OCP has not been observed in vascular calcifications, it has been strongly suggested as a precursor phase to biological apatite found in natural and prosthetic heart valves [128,129]. In surgery, OCP is used for implantation into bone defects [130,131,132,133,134,135]. For comprehensive information on OCP, the readers are referred to a monograph [120].

### 3.6. β-TCP

β-TCP (β-tricalcium phosphate, β-Ca_3_(PO_4_)_2_; the chemically correct name is calcium phosphate tribasic beta) cannot be precipitated from aqueous solutions. It is a high temperature phase, which can only be prepared at temperatures above 800 ºC by thermal decomposition of CDHA or by solid-state interaction of acidic calcium orthophosphates, *e.g.*, DCPA, with a base, *e.g*., CaO. Apart from the chemical preparation routes, ion-substituted β-TCP can be prepared by calcining of bones: such type of β-TCP is occasionally called “bone ash”. In β-TCP, there are three types of crystallographically nonequivalent PO_4_^3-^ groups located at general points of the crystal, each type with different intratetrahedral bond lengths and angles. At temperatures above ~ 1125 ºC, β-TCP transforms into a high-temperature phase α-TCP. Being the stable phase at room temperature, β-TCP is less soluble in water than α-TCP (Table 2). Furthermore, the ideal β-TCP structure contains calcium ion vacancies that are too small to accommodate calcium ions, but allow for the inclusion of magnesium ions, which thereby stabilize the structures.

Pure β-TCP never occurs in biological calcifications. Only the Mg-substituted form called whitlockite [136] (β-TCMP – β-tricalcium magnesium phosphate, β-(Ca,Mg)_3_(PO_4_)_2_) is found in dental calculi and urinary stones, dentinal caries, salivary stones, arthritic cartilage, as well as in some soft-tissue deposits [13,46,47,48,143]. However, it has not been observed in enamel, dentin or bone. In biomedicine, β-TCP is used in calcium orthophosphate bone cements [144,145,146,147]. In combination with HA, β-TCP forms a biphasic calcium phosphate (BCP [148]) [151,152,153,154,155,156,157,158,159,160]. Both β-TCP [161] and BCP [151,152,153,154,155,156,157,158,159,160] are widely used as a bone substitution bioceramics. Pure β-TCP is added to some brands of toothpaste as a gentle polishing agent. Multivitamin complexes with calcium orthophosphate are widely available in the market and β-TCP is used as the calcium phosphate there. In addition, it serves as a texturizer, bakery improver and anti-clumping agent for dry powdered food (flour, milk powder, dried cream, cocoa powder). In addition, β-TCP is added as a dietary or mineral supplement to food and feed, where it is marked as E341 according to the European Classification of Food Additives. Occasionally, it might be used as an inert filler in pelleted drugs. Other applications comprise porcelains, pottery, enamel, using as a component for mordants and ackey, as well as a polymer stabilizer [88]. β-TCP of a technical grade (as either calcined natural phosphorites or bone dust) is used as a slow release fertilizer for acidic soils [20].

### 3.7. α-TCP

α-TCP (α-tricalcium phosphate, α-Ca_3_(PO_4_)_2_; the chemically correct name is calcium phosphate tribasic alpha) is usually prepared from β-TCP by heating above ~ 1125 ºC and it might be considered a high temperature phase of β-TCP. However, at the turn of the millennium, the previously forgotten data that the presence of silicates stabilized α-TCP at lower temperatures of 800 – 1000 ºC [162] has been rediscovered again. Such type of α-TCP is called “silicon stabilized α-TCP” [163,164,165,166,167,168].

Although α-TCP and β-TCP have exactly the same chemical composition, they differ by the crystal structure (Table 3) and solubility (Table 2). In addition, β-TCP is more stable than the α-phase [169]. Therefore, of them, α-TCP is more reactive in aqueous systems, has a higher specific energy and it can be hydrolyzed to a mixture of other calcium phosphates. It never occurs in biological calcifications but in medicine chemically pure α-TCP is used in calcium phosphate cements [85,93,94,95,96,108,109,110,170,171]. Pure α-TCP has received not much interest in the biomedical field. The disadvantage of using α-TCP is its quick resorption rate, which limits its application in this area. However, the silicon stabilized α-TCP (more precisely as a biphasic composite with HA) has been commercialized as a starting material to produce bioresorbable porous ceramic scaffolds to be used as artificial bone grafts [161,163,164,165,166,167]. Theoretical insights into bone grafting properties of the silicon-stabilized α-TCP may be found in Ref. [172]. Surface and adsorption properties of α-TCP are described in Ref. [173]. Technical grade α-TCP can be used as a fertilizer [88].

### 3.8. ACP

ACP (amorphous calcium phosphate, Ca_x_H_y_(PO_4_)_z_·nH_2_O, n = 3 – 4.5; 15 – 20% H_2_O) is often encountered as a transient phase during the formation of calcium orthophosphates in aqueous systems. Usually, ACP is the first phase precipitated from a supersaturated solution prepared by rapid mixing of solutions containing ions of calcium and orthophosphate [14,174,175,176,177,178,179]. ACP is thought to be formed at the beginning of the precipitation due to a lower surface energy than that of OCP and apatites [175]. The amorphization level of ACP increases with the concentration increasing of Ca- and PO_4_-containing solutions, as well as at a higher solution pH and a lower crystallization temperature. A continuous gentle agitation of as precipitated ACP in the mother solution, especially at elevated temperatures, results in a slow recrystallization and formation of better crystalline compounds, such as CDHA [13,14]. The lifetime of ACP in aqueous solution was reported to be a function of the presence of additive molecules and ions, pH, ionic strength and temperature. Thus, ACP may persist for appreciable periods and retain the amporphous state under some specific experimental conditions [180]. The chemical composition of ACP strongly depends on the solution pH and the concentrations of mixing solutions. For example, ACP with Ca/P ratios in the range of 1.18 (precipitated at solution pH = 6.6) to 1.53 (precipitated at solution pH = 11.7) [14,181] and even to 2.5 [13,46,47] have been described. The presence of poly(ethylene glycol) [182], ions of pyrophosphate, carbonate and/or magnesium in solution during the crystallization promotes formation of ACP and slows down its further transformation into more crystalline calcium orthophosphates, while the presence of fluoride has the opposite effect [13,14,15,63,183]. The solution-mediated transformation of ACP to CDHA, which can be described by a ”first-order” rate law, is a function only of the solution pH and depends upon the experimental conditions which regulate both the dissolution of ACP and the formation of early HA nuclei [184].

**Figure 3:**
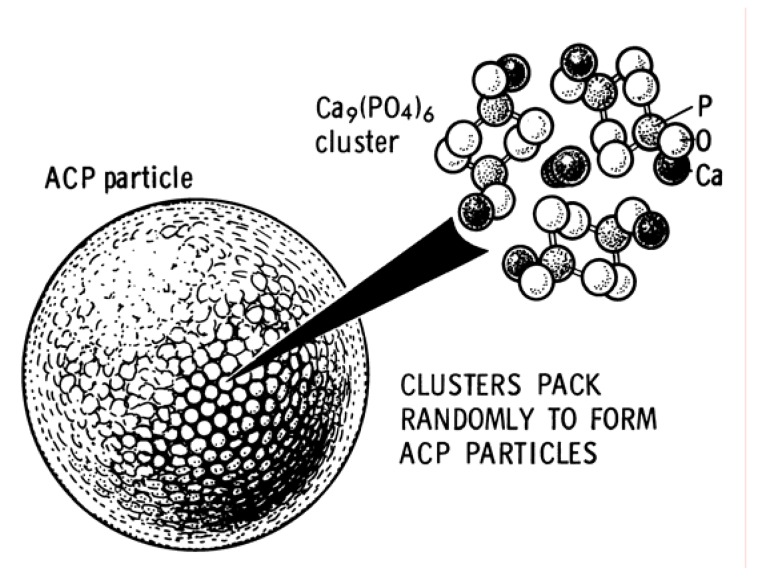
A model of ACP structure. Reprinted from Ref. [190] with permission.

As all amorphous compounds are characterized by a lack of the long-range order, it is problematic to discuss the crystal structure of ACP (it is X-ray amorphous). Concerning the short-range order in ACP, it is uncertain either, because it depends on the preparation conditions, storage, admixtures, *etc*. It is well known that ACP contains 10 – 20% by weight of tightly bound water, which is removed by vacuum drying at elevated temperature [185]. Infrared spectra of ACP show broad featureless phosphate absorption bands. Electron microscopy of ACP usually shows featureless nearly spherical particles with diameters in the range of 20 to 200 nm. However, there is a questionable opinion that ACP has an apatitic structure but with a crystal size so small, that it is X-ray amorphous. This is supported by X-ray absorption spectroscopic data (EXAFS) on biogenic and synthetic samples [186,187,188,189]. On the other hand, it was proposed that the basic structural unit of ACP is a 9.5 Å diameter, roughly spherical cluster of ions with the composition Ca_9_(PO_4_)_6_ (Figure 3) [14,181,190,191]. These clusters were found experimentally as first nuclei during the crystallization of HA and a model was developed to describe the crystallization of HA as a step-wise assembly of these units [192] (see HA below). Biologically, ACP (often containing ions of Na, Mg, carbonate and pyrophosphate) is found in soft-tissue pathological calcifications (*e.g.*, heart valve calcifications of uremic patients) [13,46,47,48]. In medicine, pure ACP is used in calcium orthophosphate cements [93,94,95] and as a filling material in dentistry. Bioactive composites of ACP with polymers have properties suitable for use in dentistry [193,194,195,196] and surgery [197,198,199,200]. Due to a reasonable solubility and physiological pH of aqueous solutions, ACP appeared to be consumable by some microorganisms and, due to this reason, it might be added as a mineral supplement to culture media. Non-biomedical applications of ACP comprise its using as a component for mordants and ackey. In food industry, ACP is used for syrup clarification. Occasionally, it is used as inert filler in pelleted drugs. In addition, ACP is used in glass and pottery production and as a raw material for production of some organic phosphates. For further details on ACP, interested readers are referred to specialized reviews [191,201].

### 3.9. CDHA

CDHA (calcium-deficient hydroxyapatite, Ca_10-*x*_(HPO_4_)_*x*_(PO_4_)_6-*x*_(OH)_2-*x*_ (0 < *x* < 1)) can be easily prepared by simultaneous addition of calcium- and orthophosphate-containing solutions into boiling water, followed by boiling the suspension for several hours. During this time, the initially precipitated ACP is restructured and transformed into CDHA [202]. Therefore, there are many similarities in the structure, properties and application between the precipitated in alkaline solutions (pH > 8) ACP and CDHA. Recent data indicated on presence of intermediate phases during further hydrolysis of CDHA to a more stable HA-like phase [206]. CDHA crystals are poorly crystalline and of submicron dimensions. It has a very large specific surface area, typically 25 – 100 m^2^/g. On heating above 700 ºC, dry CDHA with Ca/P = 1.5 will convert to β-TCP and that with 1.5 < Ca/P < 1.67 will convert into a mixture of HA and β-TCP (the above-mentioned BCP) [151,152,153,154,155,156,157,158,159]. A reasonable solid-state mechanism of a high-temperature transformation of CDHA into BCP has been proposed [207,208].

The variability in Ca/P molar ratio of CDHA has been explained through different models: surface adsorption, lattice substitution and intercrystalline mixtures of HA and OCP [209]. Due to a lack of stoichiometry, CDHA usually contains other ions [45]. The extent depends on the counter-ions of the chemicals used for preparation (*e.g.*, Na^+^, Cl^-^). Direct determinations of the CDHA structures are still missing and the unit cell parameters remain uncertain. However, the long-range order exists and the following lattice parameters have been reported for formate (HCO_2_^-^) containing CDHA with Ca/P = 1.596 (ionic): *a* = 9.4729(20) and *c* = 6.8855(9) Å. Ca^2+^ ions were lost exclusively from Ca2 sites, while the PO_4_ tetrahedron volume and P – O bonds were 4.4% and 1.4% smaller, respectively, than those in HA [210].

A systematic study of defect constellations in CDHA is available in the literature [211]. As a first approximation, CDHA may be considered as HA with some ions missing [212]. The more calcium is deficient, the more disorder and imperfections are in CDHA structure [213]. According to the chemical formula of CDHA (Table 2), there are vacancies of Ca^2+^ (mainly on Ca2 sites) and OH^-^ ions in crystal structure of this compound [210,211,212,213,214,215,216]. However, nothing is known about the vacancies of orthophosphate ions: in CDHA, a portion of PO_4_^3-^ ions is either protonated (as HPO_4_^2-^) or substituted by other ions (*e.g.*, CO_3_^2-^) [217]. Theoretical investigations of the defect formation mechanism relevant to non-stoichiometry in CDHA are available elsewhere [218].

Unsubstituted CDHA (*i.e.* containing ions of Ca^2+^, PO_4_^3-^, HPO_4_^2-^ and OH^-^ only) does not exist in biological systems. The ion substituted CDHA: Na^+^, K^+^, Mg^2+^, Sr^2+^ for Ca^2+^; CO_3_^2-^ for PO_4_^3-^ or HPO_4_^2-^; F^-^, Cl^-^, CO_3_^2-^ for OH^-^, plus some water forms biological apatite – the main inorganic part of animal and human normal and pathological calcifications [13,45,46]. Therefore, CDHA is a very promising compound for industrial manufacturing of artificial bone substitutes. Non-biomedical applications of CDHA are similar to those of ACP. Recently, CDHA was found to possess catalytic activity for the production of biogasoline [219].

### 3.10. HA

HA (or OHAp) (hydroxyapatite [220], Ca_5_(PO_4_)_3_(OH), but usually written as Ca_10_(PO_4_)_6_(OH)_2_ to denote that the crystal unit cell comprises two molecules) is the second most stable and least soluble calcium orthophosphate after FA. Chemically pure HA crystallizes in the monoclinic space group *P*2_1_/b [221]. However, at temperatures above 250 ºC, there is a monoclinic to hexagonal phase transition in HA (space group *P*6_3_/m) [14,74,181,222,223]. The detailed description of the HA structure was first reported in 1964 [224] and its interpretation in terms of aggregation of Ca_9_(PO_4_)_6_ clusters, the so-called Posner’s clusters, has been widely used since publication of the article by Posner and Betts [185]. The Ca_9_(PO_4_)_6_ clusters appeared to be energetically favored in comparison to alternative candidates including Ca_3_(PO_4_)_2_ and Ca_6_(PO_4_)_4_ clusters [225]. In hexagonal HA, the hydroxide ions are more disordered within each row, when compared with the monoclinic form, pointing either upward or downward in the structure. This induces strains that are compensated for by substitutions or ion vacancies. Some impurities, like partial substitution of hydroxide by fluoride or chloride, stabilize the hexagonal structure of HA at ambient temperature. Due to this reason, hexagonal HA is seldom the stoichiometric phase and very rare single crystals of natural HA always exhibit the hexagonal space group. The hexagonal structure of HA is a more common one for biomedical applications. The crystal structure of HA is well described elsewhere [14,73,74,75], the detailed analysis of the electronic structure, bonding, charge transfer and optical properties are also available [226,227], while the readers interested in Posner’s clusters are referred to other papers [225,228,229,230]. A shell model has been developed to study the lattice dynamics of HA [231].

Several techniques may be utilized for HA preparation; they can be divided into solid-state reactions and wet methods [232], which include precipitation, hydrothermal and hydrolysis of other calcium orthophosphates. Even under the ideal stoichiometric conditions, the precipitates are generally non-stoichiometric, suggesting intermediate formation of precursor phases. HA can be prepared in aqueous solutions by mixing exactly stoichiometric quantities of Ca- and PO_4_-containing solutions at pH > 9, followed by boiling for several days in CO_2_-free atmosphere (the ageing or maturation stage), filtration, drying and, usually, sintering at about 1000 ºC [233]. As the first precipitates are rich in non-apatitic environments (see ACP and CDHA), the ageing stage appears to be very important: the Ca/P molar ratio of 1.67 was found to be attained in as little as 5 hours after the completion of the reaction at 90°C [234]. The surface of freshly precipitated HA is composed of a structured hydrated layer containing easily exchangeable mobile ionic species [235]. Usually unsintered HA is poorly crystalline and often non-stoichiometric, resembling the aforementioned CDHA. However, highly crystalline HA can be prepared from an aqueous solution [236]. Microcrystalline samples of HA can also be prepared by solid-state reaction of other calcium phosphates (*e.g*., MCPM, DCPA, DCPD, OCP) with CaO, Ca(OH)_2_, or CaCO_3_ at temperatures above 1200 ºC in an atmosphere of equal volumes of water and nitrogen. HA can be prepared by hydrothermal synthesis [14,181,237]. A water-free synthesis can be performed in ethanol from Ca(OEt)_2_ (Et = ethyl) and H_3_PO_4_ [238,239]. In addition, HA can be prepared by mechanochemical synthesis of a dry mixture of CaO and DCPD [232,240] or from coral skeletal carbonate by hydrothermal exchange [241,242,243]. Relatively large single crystals of HA might be prepared from those of chlorapatite [244] or by recently developed controlled homogeneous precipitation method [245]. Lower sized particles of HA might be prepared by a pyrosol technique, where an aerosol, containing calcium and orthophosphate ions in the adequate ratio, is transported to a furnace where the pyrolisis takes place [246]. Synthesis of nanosized HA has also been described [247,248], while the chronological development of nanosized HA synthesis can be found in another paper [249]. Two-dimensional nanocrystalline HA may be also synthesized [250]. Space-grown and terrestrial HA crystals were found to differ in size: the former appeared to be at least 1 – 1.5 orders of magnitude bigger in length [251,252]. Transparent HA ceramics can be prepared as well [253,254,255,256]. Detailed information on HA synthesis is available elsewhere [257,258,259,260,261,262,263]. In addition, there are good reviews on HA solubility, crystal growth and intermediate phases of HA crystallization [264], as well as on HA dissolution [265]. The electronic and crystallographic structures of apatites can be found in another paper [226].

Pure HA never occurs in biological systems. However, due to the chemical similarities to bone and teeth mineral (Table 1), HA is widely used as a coating on orthopedic (*e.g.*, hip joint prosthesis) and dental implants [266,267,268,269,270,271,272]. HA particles might be implanted as well [273]. Due to a great similarity to biological apatite, HA has been used for a long time in liquid chromatography of nucleic acids, proteins and other biological compounds [274,275,276,277,278,279,280,281] and for drug delivery purposes [282,283]. Also, HA is added to some brands of toothpaste as a gentle polishing agent instead of calcium carbonate. Besides, it can be used as an environmentally friendly filler for elastomers [284], a sorbent of poisonous chemical elements [285] and a carrier for catalysts [286,287]. To conclude this topic, one should mention some other reviews devoted to HA and its biomedical applications [288,289,290,291,292,293].

### 3.11. FA

**Figure 4:**
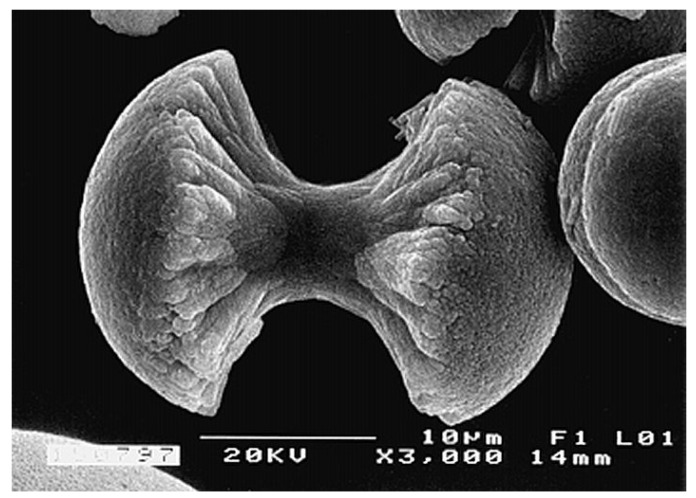
A biomimetically grown aggregate of FA that was crystallized in a gelatin matrix. Its shape can be explained and simulated by a fractal growth mechanism. Scale bar: 10 μm (taken from Ref. [295] with permission).

FA (or FAp) (fluorapatite, Ca_5_(PO_4_)_3_F, but is usually written as Ca_10_(PO_4_)_6_F_2_ to denote that the crystal unit cell comprises two molecules) is the hardest (5 according to the Mohs’ scale of mineral hardness), most stable and least soluble compound among all calcium orthophosphates (Table 2). Perhaps, such “extreme” properties of FA are related to the specific position of F^-^ ions in the center of Ca2 triangles of the crystal structure [74]. Due to its properties, FA is the only calcium orthophosphate that naturally forms large deposits suitable for the commercial use [19,20,21,22] (see also Figure 1). Preparation techniques for chemically pure FA are similar to the aforementioned ones for HA, but the synthesis must be performed in presence of the necessary amount of F^-^ ions (usually, NaF or NH_4_F is added). Unlike that for HA (see CDHA), no data are available on calcium-deficient FA. Under some special crystallization conditions, FA might form unusual dumbbell-like fractal morphology that finally closed to spheres (Figure 4) [294,295,296,297,298,299]. A hierarchical structure for FA was proposed [300]. The crystal structure of FA for the first time was studied in 1930 [301,302] and is well described elsewhere [14,73,74,75,303]. The detailed analysis of the electronic structure, bonding, charge transfer and optical properties is available as well [227]. In addition, there are reviews on FA solubility [264] and the dissolution mechanism [265].

FA easily forms solid solutions with HA with any desired F/OH molar ratio. Such compounds are called fluorhydroxyapatites (FHA) or hydroxyfluorapatites (HFA) and described with a chemical formula Ca_10_(PO_4_)_6_(OH)_2-*x*_F_*x*_, where 0 < *x* < 2. If the F/OH ratio is either uncertain or not important, the chemical formula of FHA and HFA is often written as Ca_10_(PO_4_)_6_(F,OH)_2_. The lattice parameters, crystal structure, solubility and other properties of FHA and HFA lay in between those for the chemically pure FA and HA [304,305,306,307,308]. 

Like pure HA, pure FA never occurs in biological systems. Obviously, a lack of the necessary amount of toxic fluorides (the acute toxic dose of fluoride is ~ 5 mg/kg of body weight) in living organisms is the main reason of this fact (pure FA contains 3.7 % mass. F). Shark teeth enameloid [63,309,310,311,312,313,314] and some exoskeletons of mollusks [315] seem to be the only exceptions because they contain substantial amounts of FA. Among all normal calcified tissues of humans, the highest concentration of fluorides is found in bones and the lowest – in dental enamel [316] (Table 1). However, even in bones, the total amount of fluorides is not enough to form FA; it is generally considered that the inorganic part of bones consists of ion-substituted CDHA. Due to the lowest solubility, good chemical stability and toxicity of high amounts of fluorides, chemically pure FA is rarely used as a bone substituting material [323]. However, due to the ability to form FHA and/or HFA, minor amounts of fluorides might be intentionally added to calcium orthophosphate biomaterials [324,325,326,327,328,329,330]. The effect of fluoride contents in FHA on both osteoblast behavior [331] and leukemia cells proliferation [332] has been described.

### 3.12. TTCP

TTCP (or TetCP) (tetracalcium phosphate or tetracalcium phosphate monoxide Ca_4_(PO_4_)_2_O; the mineral hilgenstockite [333]) is the most basic calcium orthophosphate. However, its solubility in water is higher than that of HA (Table 2). TTCP cannot be precipitated from aqueous solutions. It can be prepared only by a solid-state reaction above 1300 ºC, *e.g.*, by heating homogenized equimolar quantities of DCPA and CaCO_3_ in dry air, or in a flow of dry nitrogen [14,181,336]. DCPA might be replaced by ammonium orthophosphates [337]. These reactions should be carried out in a dry atmosphere, under vacuum or with rapid cooling (to prevent uptake of water and formation of HA). TTCP is not very stable in aqueous solutions: it slowly hydrolyses to HA and calcium hydroxide [14,181] and consequently, TTCP is never found in biological calcifications. In medicine, TTCP is widely used for preparation of various self-setting calcium phosphate cements [78,86,93,106,338]; however, to the best of my knowledge, there is no commercial bone-substituting product consisting solely of TTCP.

There is an opinion [74], that the aforementioned calcium orthophosphates might be classified into three major structural types: (i) the apatite type, Ca_10_(PO_4_)_6_X_2_, which includes HA, FA, CDHA, OCP and TTCP; (ii) the glaserite type, named after the mineral glaserite, K_3_Na(SO_4_)_2_, which includes all polymorphs of TCP and, perhaps, ACP; (iii) the Ca – PO_4_ sheet-containing compounds, which include DCPD, DCPA, MCPM and MCPA. According to the authors, a closer examination of the structures revealed that all available calcium orthophosphates could be included into distorted glaserite type structures, but with varying degrees of distortion [74].

### 3.13. Substituted Calcium Orthophosphates

To conclude this part, one should briefly mention carbonateapatite [339,340,341,342,343], chlorapatite [344,345] and various ion-substituted calcium orthophosphates [45,346]. Usually, they are of a non-stoichiometric nature and there are too many of them to be mentioned in one review; therefore, the readers are referred to books and monographs covering the subject [13,14,15,19,21,27,63,181,289,293]. In addition, there is a very good review, in which the structures of more than 75 chemically different apatites have been discussed [73].

It is interesting to note, that chemical elements not found in natural bones can be intentionally incorporated into calcium orthophosphate biomaterials to produce special properties. For example, addition of Ag^+^ [347,348], Zn^2+^ and Cu^2+^ [348] has been used for imparting antimicrobial effects, while radioactive isotopes of ^90^Y [349], ^153^Sm [350,351,352] and ^186^Re [350] have been incorporated into HA bioceramics and injected into knee joints to treat rheumatoid joint synovitis [349,350,352]. More to the point, apatites were found to be able to incorporate individual molecules, such as water, oxygen and carbon dioxide [45].

## 4. Biological Calcium Orthophosphate Hard Tissues 

**Figure 5:**
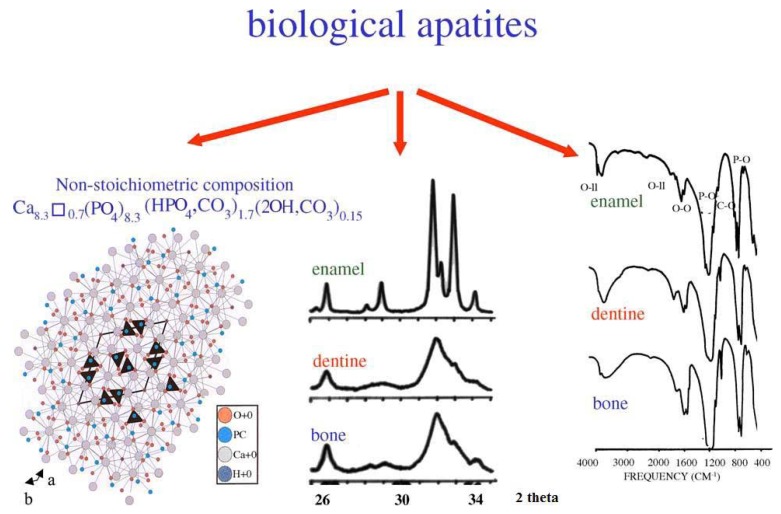
Crystal structure of biological apatites. Powder X-ray diffraction patterns and infrared spectra of enamel, dentine and bone. Reprinted from Ref. [355] with permission.

Biological mineralization (biomineralization) is the process of *in vivo* formation of inorganic minerals [311,312]. As shown in Table 1 and discussed above, in the bodies of mammals the vast majority of both normal and pathological calcifications consist of ion-substituted calcium orthophosphates, mainly of apatitic structure [50,353]. On an element scale, bone apatite nanocrystals exhibit a variety of substitutions and vacancies that make the Ca/P molar ratio diverge from the stoichiometric HA ratio of 1.67. The impurities in biological apatite of bones and teeth introduce significant stresses into the crystal structure, which make it less stable and more reactive. Among all substituting ions, the presence of 4 – 8% of carbonates instead of orthophosphate anions (so called, B-type substitution [13,14,15,343]) and of 0.5 – 1.5 % of Mg is of the special importance because it leads to large lattice strain and significantly increases the solubility [353,354]. High concentrations of magnesium and carbonates in bone or dentin compared with enamel (Table 1) may explain a higher solubility and a lower crystallinity (smaller crystal size) of bone or dentin compared with enamel. In addition, the crystals of biological apatite are always very small, which also increases its solubility when compared with that for the chemically pure HA and even CDHA [45]. Small dimensions and a low crystallinity are two distinct features of biological apatite, which, combined with their non-stoichiometric composition, inner crystalline disorder and presence of other ions in the crystal lattice, allow explaining their special behavior. For example, the small crystal size means that a large percentage of the atoms are on the surface of the crystal, providing a large specific surface area for sorption of ions, proteins and drugs [354,355]. The major properties of biological apatite are summarized in Figure 5. It is interesting to note, that the solubility and equilibrium phenomena of calcium orthophosphates related to the calcification process have been studied, at least, since 1925 [356,357].

The calcium orthophosphate nature of bones was first determined in 1913 [358]. This discovery was clarified afterwards, suggesting that the bone mineral could be carbonated apatite [359,360]. Further optical and X-ray analysis of bones and other mineralized tissues matched analyses of two apatites: FA and dahllite [361]. Additional historical data on this point are available in literature [44]. Nowadays, according to Weiner and Wagner: “the term bone refers to a family of materials, all of which are built up of mineralized collagen fibrils” [362,363]. For mammals, this family of materials includes dentin – the material that constitutes the inner layers of teeth, cementum – the thin layer that binds the roots of teeth to the jaw, deer antlers and some other materials [362,364]. It is worth noting, that bones and teeth contain almost 99% of the total body calcium and about 85% of the total body phosphorus that amounts to a combined mass of approximately 2 kg in an average person [365,366]. In addition, it is important to recognize that calcium orthophosphates of bones are by no means inert; they play an important role in the metabolic functions of the body. The recent data on the physico-chemical and crystallographic study of biological apatite have been reviewed elsewhere [367]. Besides, there is a comprehensive review on the application of surface science methods to study the properties of dental materials and related biomaterials [368].

### 4.1. Bone

Bone (Latin: *os*), also called osseous tissue, is a type of hard endoskeletal connective tissue found in many vertebrate animals. All bones of a single animal are, collectively, known as the skeleton. True bones are present in bony fish (osteichthyes) and all tetrapods. Bones support body structures, protect internal organs and, in conjunction with muscles, facilitate movement [369]. In addition, bones are also involved with blood cell formation, calcium metabolism and act for mineral storage. From the material point of view, bone is a dynamic, highly vascularized tissue that is formed from a complicated composite containing both inorganic (Table 1) and biooorganic compounds (chiefly, collagen) [353,370,371,372,373,374,375,376]. The inorganic to biooorganic ratio is approximately 75% to 25% by dry weight and about 65% to 35% by volume. This ratio not only differs among animals, among bones in the same animal and over time in the same animal, but also it exerts a major control over the material properties of bone, such as its toughness, ultimate strength and stiffness. In general, load-bearing ability of bones depends on not only architectural properties, such as cortical thickness and bone diameter, but also intrinsic, size-independent, material properties such as porosity, level of mineralization, crystal size and properties derived from the organic phase of bone [377]. A higher mineral to collagen ratio typically yields stronger, but more brittle, bones [378,379,380]. For example, bone from the leg of a cow has a relatively high concentration of calcium orthophosphates (for support), whereas bone from the antler of a deer has a relatively high concentration of collagen (for flexibility) [80]. It is interesting to note, that bone exhibits several physical properties such as piezoelectricity [381] and pyroelectricity [382].

**Figure 6:**
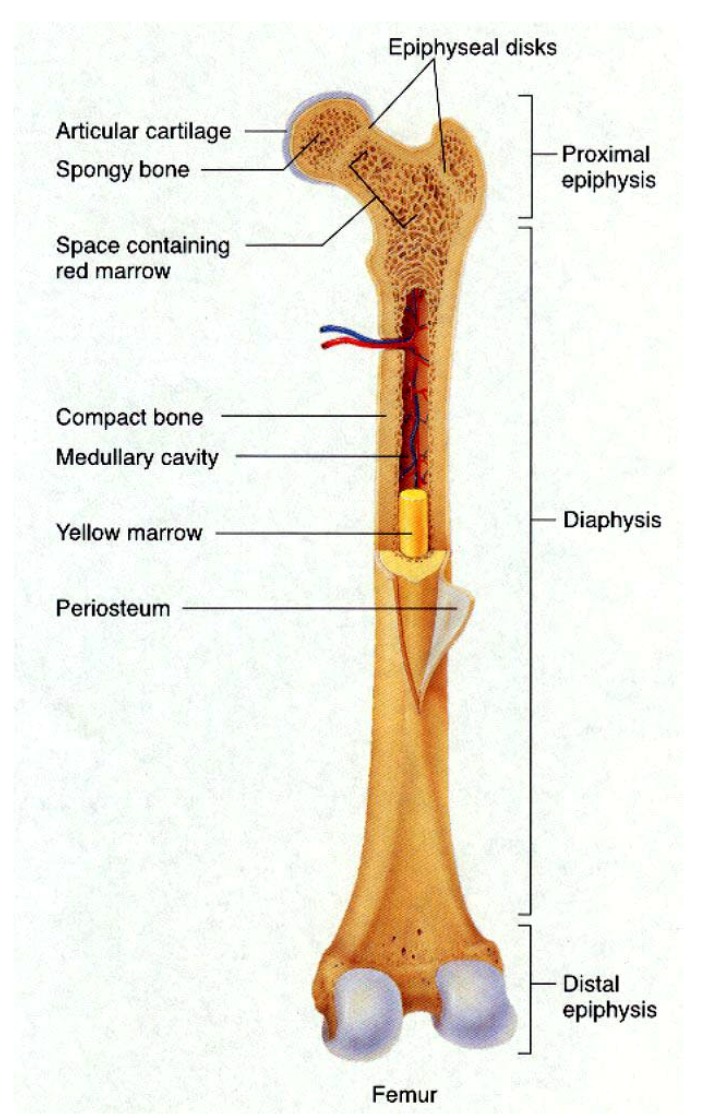
General structure of a mammalian bone. Other very good graphical sketches of the mammalian bone structure are available in Refs. [50,355].

Stability of the mineral composition of bones has a very long history: calcium orthophosphates were found in dinosaur fossils [31,383,384,385,386]. Therefore, organisms have had a great deal of time to exploit the feedback between composition and structure in apatite, on the one hand, and benefit from its biological functionality, on the other. Bones of modern animals is a relatively hard and lightweight porous composite material, formed mostly of biological apatite (*i.e.*, CDHA with ionic substitutions). It has relatively high compressive strength but poor tensile strength [387]. While bone is essentially brittle, it has a degree of significant plasticity contributed by its organic components. Usually bone is composed of a relatively dense outer layer (cortical or compact bone) covering an internal mesh-like structure (average porosity of 75 – 95%) of cancellous (other terms: spongy, trabecular) bone, the density of which is about 0.2 g/cm^3^ but it may vary at different points (Figure 6). The porosity reduces the strength of bones but also reduces their weight.

Cortical bone makes up a large portion of skeletal mass; but due to its high density (~ 1.80 g/cm^3^) it has a low surface area. Cancellous bone has an open meshwork or honeycomb-like structure. It has a relatively high surface area but forms a smaller portion of the skeleton. Bone is a porous material with the pore sizes range from 1 to 100 μm in normal cortical bone and 200 to 400 μm in trabecular bone. 55 to 70% of the pores in trabecular bone are interconnected [13,46,47,61,62,63,311,362,371,372,373,374,375,388,389,390,391].

**Figure 7:**
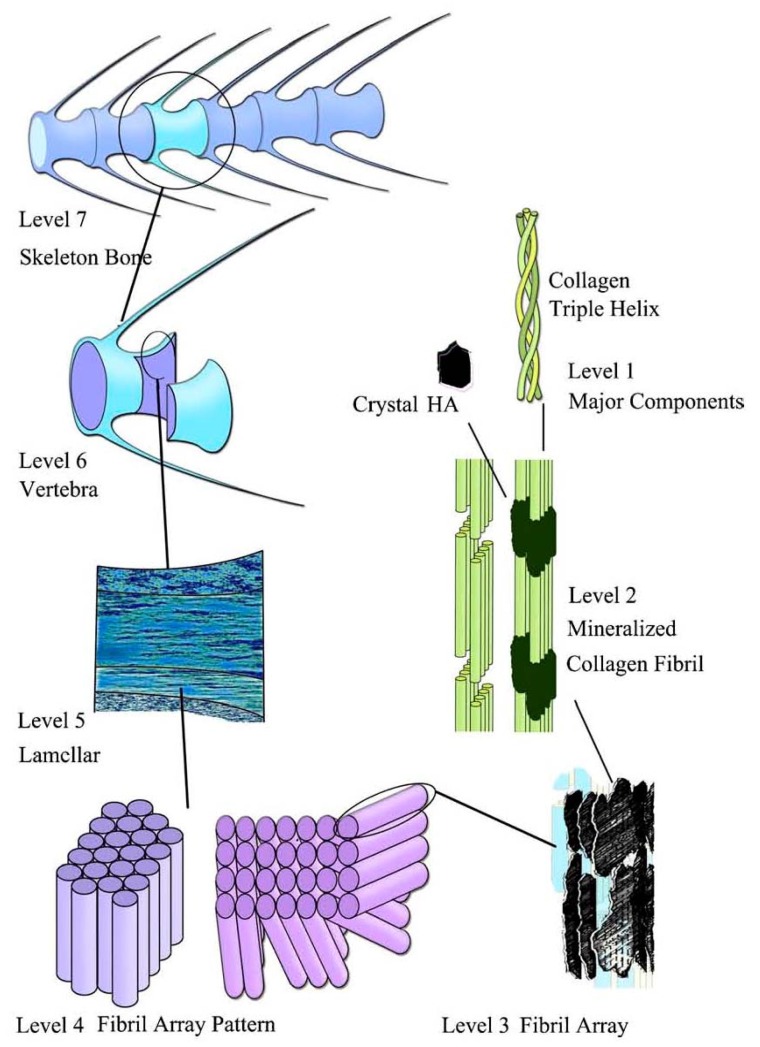
The seven hierarchical levels of organization of the zebrafish skeleton bone. Level 1: Isolated crystals and part of a collagen fibril with the triple helix structure. Level 2: Mineralized collagen fibrils. Level 3: The array of mineralized collagen fibrils with a cross-striation periodicity of nearly 60-70 nm. Level 4: Two fibril array patterns of organization as found in the zebrafish skeleton bone. Level 5: The lamellar structure in one vertebra. Level 6: A vertebra. Level 7: Skeleton bone. Reprinted from Ref. [418] with permission. Other good graphical sketches of the hierarchical structure of bones are available in Refs. [362,400].

Bone can be either woven or lamellar. The fibers of woven bone are randomly aligned and as the result have a low strength. In contrast, lamellar bone has parallel fibers and is much stronger. Woven bone is put down rapidly during growth or repair [392] but as growth continues, it is often replaced by lamellar bone. The replacement process is called “secondary bone formation” and described in detail elsewhere [393 and references therein]. In addition, bones might be long, short, flat and irregular. The sizes and shapes of bones reflect their function. Namely, broad and flat bones, such as scapulae, anchor large muscle masses, flat skull bones protect the brain, ribs protect the lungs, pelvis protects other internal organs, short tubular bones in the digits of hands and feet provide specific grasping functions, hollow and thick-walled tubular bones, such as femur or radius, support weight and long bones enable locomotion [394,395]. Long bones are tubular in structure (*e.g.*, the tibia). The central shaft of a long bone is called the diaphysis and has a medullar cavity filled with bone marrow (Figure 6). Surrounding the medullar cavity is a thin layer of cancellous bone that also contains marrow. The extremities of the bone are called the epiphyses and are mostly cancellous bone covered by a relatively thin layer of compact bone. Short bones (*e.g.*, finger bones) have a similar structure to long bones, except that they have no medullar cavity. Flat bones (*e.g.*, the skull and ribs) consist of two layers of compact bone with a zone of cancellous bone sandwiched between them. Irregular bones (*e.g.*, vertebrae) do not conform to any of the previous forms. Thus, bones are shaped in such a manner that strength is provided only where it is needed. All bones contain living cells embedded in a mineralized organic matrix that makes up the main bone material [394,395,396]. The structure of bone is most easily understood by differentiating between seven levels of organization because bone exhibits a strongly hierarchical structure (Figure 7) [289,311,353,362,370,371,372,373,374,375,381,382,383,384,385,386,388,389,390,391,397,398,399].

The mechanical properties of bone reconcile high stiffness and high elasticity in a manner that is not yet possible with synthetic materials [400]. Cortical bone specimens have been found to have tensile strength in the range of 78.8 – 151.0 MPa in longitudinal direction and 51.0 – 56.0 MPa in transversal direction. Bone’s elasticity is also important for its function giving the ability to the skeleton to withstand impact. Estimates of modulus of elasticity of bone samples are of the order of 17.0 – 20.0 GPa in longitudinal direction and of 6.0 – 13.0 GPa in the transversal direction [401]. The elastic properties of bone were successfully modeled at the level of mineralized collagen fibrils via step-by-step homogenization from the staggered arrangement of collagen molecules up to an array of parallel mineralized fibrils [402]. Recent investigations revealed that bone deformation was not homogeneous but distributed between a tensile deformation of the fibrils and a shearing in the interfibrillar matrix between them [403,404]. Furthermore, there is a good review on the effects of the microscopic and nanoscale structure on bone fragility [405].

Nanoscopically, the constituting building blocks of bone are mineralized collagen fibrils of 80 to 100 nm thickness and a length of a few to tens of microns. These are composites of biological apatite and molecules of type I collagen [50,362,370,376,406]. Some evidence for direct physical bonding between the collagen fibers and apatite crystals in bone has been found [407]. Eppell *et al*. used atomic force microscopy to measure the crystallites of mature cow bone [408]. They are always platelet-like (elongated along the crystallographic *c*-axis) and very thin [49,409,410,411], with remarkably uniform thicknesses (determined in transmission electron microscopy) of 2 – 4 nm [412] (just a few unit cells thick – see Table 1). The nanocrystals of biological apatite exist in bones not as discrete aggregates but rather as a continuous phase, which is indirectly evidenced by a very good strength of bones. This results in a very large surface area facing extracellular fluids, which is critically important for the rapid exchange of ions with these fluids. The nanocrystals of biological apatite are inserted in a nearly parallel way into the collagen fibrils, while the latter are formed by self-assembly [413] of collagen triple helices [362,370,415,416,417,418] using the self-organization mechanism [419,420]. Recent data from electron diffraction studies revealed that that the mineral plates of biological apatite are not quite as ordered as previously assumed [393]. This imperfect arrangement of nearly parallel crystals has been supported by recent SAXS and transmission electron microscopy studies [421].

The lowest level of hierarchical organization of bone has been simulated by CDHA precipitation on peptide-amphiphile nanofibers [420]. However, apatite platelets nucleating on the surface of peptide tubules are not similar to the nanostructure of bone and they are only an example of surface induced nucleation (and not accurately characterized either), while the nanostructure of bone consists of intra-fibrillar platelets intercalated *within* the collagen fibrils. Olszta and Gower were the first to truly duplicate the bone nanostructure [393]. Unfortunately, the interface between collagen and crystals of biological apatite is still poorly understood; for the available details, the readers are referred to a review devoted to the structure and mechanical quality of the collagen/mineral nano-composite of bones [406]. There is still no clear idea why the crystals of biological apatite are platelet-shaped even though dahllite has hexagonal crystal symmetry [311,362,371,372,373,374,375,381,382,383,384,385,386,388,389,390,391]. One possible reason is that they grow via an OCP transition phase, which crystals are plate-shaped [362].

The processes of bone formation (ossification) and growth are very complicated ones and it is difficult to describe them without making a deep invasion into biology. It has been studied for decades [392] but still there are missing points. Briefly, it is considered that bones appear and grow as the result of calcification (or biomineralization) of connective tissues, mainly cartilage [353,393]. The ossified tissue is invaginated with blood vessels, which bring ions of calcium and orthophosphate to be deposited in the ossifying tissue. The biomineralization process is controlled to some extent by cells and the organic matrices made by those cells facilitate the deposition of crystals [396]. There is an opinion, that, initially, the mineral crystals are formed in an environment rich in the so-called SIBLING (Small Integrin-Binding LIgand N-linked Glycoprotein) proteins. As bone crystals grow, there is greater association with proteins, such as osteocalcin, that regulate remodeling [422]. Thus, *in vivo* formation of hard tissues always occurs by mineral reinforcement of the previously formed network of soft tissues [353,393,394,395,418].

Cartilage is composed of cells (chondrocytes and their precursor forms known as chondroblasts), fibers (collagen and elastic fibers) and extracellular matrix (proteoglycans, which are a special class of heavily glycosylated glycoproteins) [423,424,425]. The initial stage involves the synthesis and extracellular assembly of the collagen matrix framework of fibrils. At the second stage, the chondrocytes calcify the matrix before undergoing the programmed cell death (apoptosis). At this point, blood vessels penetrate this calcified matrix, bringing in osteoblasts, which use the calcified cartilage matrix as a template to build bone, thus completing ossification [423,424,425].

During ossification, the crystals of biological apatite grow with a specific crystalline orientation – the *c*-axes of the crystals are roughly parallel to the long axes of the collagen fibrils within which they are deposited [353,362,364,365,366,367,368,371,372,373,376,393]. Earlier, it was believed that this process occurred via epitaxial growth mechanism [426]. The same was suggested for dentin and enamel [427,428] (see below), as well as for more primitive living organisms. For example, in the shell of the fossil marine animal *Lingula brachiopod unguis* that consists of a biological apatite, the crystal *c*-axes are oriented parallel to the β-chitin fibrils [315,429,430,431,432]. Therefore, the orientation of biological apatite crystals parallel to the long axes of the organic framework could be a general feature of calcium orthophosphate biomineralization. However, the degree of biological apatite orientation appears to be a useful parameter to evaluate *in vivo* stress distribution, nano-scale microstructure and the related mechanical function, the regenerative process of the regenerated bone and to diagnose bone diseases such as osteoarthritis [433,434]. It is interesting to note, that contrary to what might be expected in accordance with possible processes of dissolution, formation and remineralization of hard tissues, no changes in phase composition of mineral part, crystal sizes (length, width and thickness) and arrangement of crystals on collagen fibers were detected in abnormal (osteoporotic) human bones compared to the normal ones [435].

Some animals, such as newts, are able to regenerate amputated limbs. This is, of course, of high interest in regenerative medicine. Bone regeneration in the forelimbs of mature newts was studied by noninvasive X-ray microtomography to image regenerating limbs from 37 to 85 days. The missing limb skeletal elements were restored in a proximal-to-distal direction, which reiterated the developmental patterning program. However, in contrast to this proximal-distal sequence, the portion of the humerus distal to the amputation site was found to fail to ossify in synchrony with the regenerating radius and ulna. This finding suggests that the replacement of cartilage with mineralized bone close to the amputation site is delayed with respect to other regenerating skeletal elements [436].

Unlike other mineralized tissues, bone continuously undergoes a remodeling process, as it is resorbed by specialized cells called osteoclasts and formed by another type of cells called osteoblasts (so called “bone lining cells”) in a delicate equilibrium [353,393,396,437,438]. The purpose of remodeling is the release of calcium and the repair of micro-damaged bones from everyday stress. Osteoblasts are mononuclear cells primarily responsible for bone formation. They contain alkaline phosphatase, which enzymatically produces orthophosphate anions needed for the mineralization. In addition, there is one more type of the cells called osteocytes that originate from osteoblasts, which have migrated into, become trapped and surrounded by bone matrix, which they themselves produce [353,371,372,373,374,393,394,395,396].

If osteoblasts are bone-forming cells, osteoclasts are multinuclear, macrophage-like cells, which can be described as bone destroying cells because they mature and migrate to discrete bone surfaces [396,437,438]. Upon arrival, active enzymes, such as acid phosphatase, are secreted against the mineral substrate that causes dissolution. This process, called bone resorption, allows stored calcium to be released into systemic circulation and is an important process in regulating calcium balance [437,438]. The iteration of remodeling events at the cellular level is influential on shaping and sculpting the skeleton both during growth and afterwards. That is why mature bone consists of a very complex mesh of bone patches, each of which has both a slightly different structure and a different age [311,353,362,364,365,366,367,368,371,372,373,393]. The interested readers are suggested to read a review on the interaction between biomaterials and osteoclasts [439].

There is still no general agreement on the chemical mechanism of bone formation. It is clear that the inorganic part of bone consists of biological apatite, *i.e*. CDHA with ionic substitutions but without the detectable amounts of hydroxide [440,441,442,443,444]. However, the recent results of solid-state nuclear magnetic resonance on fresh-frozen and ground whole bones of several mammalian species revealed that the bone crystal OH^-^ was readily detectable; a rough estimate yielded an OH^-^ content of human cortical bone of about 20% of the amount expected in stoichiometric HA [445]. Various *in vitro* experiments on precipitation of CDHA and HA revealed that none of these compounds is directly precipitated from supersaturated aqueous solutions containing calcium and orthophosphate ions: some intermediate phases (precursors) are always involved [13,46,47,124,125,126,127,128,129,174,175,176,177,178]. Depending on the both solution pH and crystallization conditions, three calcium orthophosphates (DCPD, ACP and OCP) are discussed as possible precursors of CDHA precipitation *in vitro*. For this reason, the same calcium orthophosphates are suggested as the precursors of biological apatite formation *in vivo*.

The transient nature of the precursor phase of bone, if it exists at all, makes it very difficult to detect, especially *in vivo* [446]. However, in 1966 W. E. Brown proposed that OCP was the initial precipitate that then acted as a template upon which biological apatite nucleates [123]. This idea was extended in his further investigations [447,448,449,450]. The principal support for this concept derived from the following: (i) the close structural similarity of OCP and HA [121,122]; (ii) formation of interlayered single crystals of OCP and HA (pseudomorphs of OCP); (iii) the easier precipitation of OCP compared with HA; (iv) the apparent plate- or lath-like habit of biological apatites that does not conform to hexagonal symmetry, but looks like a pseudomorph of triclinic OCP; (v) the presence of HPO_4_^2-^ in bone mineral, particularly in newly formed bones [367]. Some evidences supporting this idea were found using high-resolution transmission electron microscopy: computer-simulated lattice images of the “central dark line” in mineralized tissues revealed that it consisted of OCP [124,125,126]. Recently, Raman spectroscopic indication for an OCP precursor phase was found during intramembranous bone formation [451]. Other evidences of OCP to HA transformation, including a mechanistic model for central dark line formation, may be found in the literature [452].

Simultaneously with Brown, the research group led by Posner proposed that ACP was the initially precipitated phase of bone and dentin mineral formation *in vivo*, thus explaining the non-stoichiometric Ca/P ratio in bones and teeth [453,454,455]. This conclusion was drawn from the following facts: (i) when calcium orthophosphates are prepared by rapid precipitation from aqueous solutions containing ions of calcium and orthophosphate at pH > 8.5, the initial solid phase is amorphous; (ii) mature bone mineral is composed of a mixture of ion-substituted ACP and poorly crystallized ion-substituted CDHA; (iii) early bone mineral has a lower crystallinity than mature bone and the observed improvement in crystallinity with the age of the bone mineral is a result of a progressive reduction in the ACP content [367,453,454,455,456,457,458,459,460,461]. However, there are thermodynamic data proving that the transition of freshly precipitated ACP into CDHA involves intermediate formation of OCP [462,463]. Recently the discovery of a stable amorphous calcium carbonate in sea urchin spines [464] reawakened the suggestion that a transient amorphous phase might also exist in bones [393,465,466,467,468]. Even more recently, evidence of an abundant ACP phase in the continuously forming fin bones of zebrafish was found [469]. The modern points of view on the bone formation mechanisms have been summarized in a recent excellent review [393], to which the interested readers are referred.

The maturation mechanism of bone minerals is not well established, mainly because of the difficulties involved in the nanostructural analyses of bone minerals [393,470]. Only indirect evidence for the *in vivo* bone mineral maturation is available. For example, X-ray diffraction patterns of bones from animals of different ages show that the reflections become sharper with age increasing [55,471]. This effect is more pronounced in the crystallographic *a*-axis [(310) reflections] as compared to the *c*-axis [(002) reflections] [472,473]. In addition, other changes, like an increase of Ca^2+^ content and a decrease of HPO_4_^2-^, occur in bone mineral with age [474,475,476,477]. Both the crystal sizes and carbonate content were found to increase during aging in rats and cows [475,476]. From a chemical point of view, these changes indicate to a slow transformation of poorly crystallized non-apatitic calcium orthophosphates into a better-crystallized ion-substituted CDHA [306]. While there are still many gaps in our knowledge, the researchers seem to be comfortable in stating that in all but the youngest bone and dentin, the only phase present is a highly disordered, highly substituted biological apatite.

Earlier, a debate related to the question on whether bone formation was an active or a passive biomineralization process. Briefly, an “active process” means the assembly of calcium orthophosphate nanocrystals into bones due to an activity of the suitable cells (*e.g.*, osteoblasts), *i.e.* within a matrix vesicle. Such structures have been discovered by transmission electron microscopy for bone and teeth formation [478,479]. A “passive process” does not require involvement of cells and means mineralization from supersaturated solutions with respect to the precipitation of biological apatite. In the latter case, thermodynamically, biomineralization might occur at any suitable nucleus. The collagen fibrils have a specific structure with a 67 nm periodicity and 35 – 40 nm gaps or holes between the ends of the collagen molecules where bone mineral is incorporated in the mineralized fibril [311,362,363,376,394,395]. Such a nucleation within these holes would lead to discrete crystals with a size related to the nucleating cavity in the collagen fibril. It was proposed that a temporary absence of the specific inhibitors might regulate the process of bone formation [480,481,482]. 

To conclude the bone subject, let us briefly mention on the practical application of bones. Cut and polished bones from a variety of animals are sometimes used as a starting material for jewelry and other crafts. Ground cattle bone is occasionally used as a fertilizer. In the Stone Age, bone was used to manufacture art, weapons, needles, catchers, amulets, pendants, headdresses, *etc*. Furthermore, in medicine, bones are used for bone graft substitutes, *e.g*., allografts from cadavers.

### 4.2. Teeth

Teeth (singular: tooth) are dense structures found in the jaws of many vertebrates. They have various structures to allow them to fulfill their different purposes. The primary function of teeth is to tear, smell and chew food, while for carnivores it is also a weapon. Therefore, teeth have to withstand a range of physical and chemical processes, including compressive forces (up to ~ 700 N), abrasion and chemical attack due to acidic foods or products of bacterial metabolism [368]. The roots of teeth are covered by gums. From the surface teeth are covered by enamel of up to ~ 2 mm thick at the cutting edges of the teeth, which helps to prevent cavities on the teeth. The biggest teeth of some gigantic animals (elephants, hippopotamuses, walruses, mammoths, narwhals, *etc.*) are known as tusks or ivory.

Similar to the various types of bones, there are various types of teeth. The shape of the teeth is related to the animal’s food, as well as its evolutionary descent. For example, plants are hard to digest, so herbivores have many molars for chewing. Carnivores need canines to kill and tear and since meat is easy to digest, they can swallow without the need for molars to chew the food well. Thus, the following types of teeth are known: molars (used for grinding up food), carnassials (used for slicing food), premolars (small molars), canines (used for tearing apart food) and incisors (used for cutting food). While humans only have two sets of teeth, some animals have many more: for example, sharks grow a new set of teeth every two weeks. Some other animals grow just one set during the life, while teeth of rodents grow and wear away continually through the animal gnawing, maintaining constant length [483,484].

Similar to bones, the inorganic part of teeth also consist of biological apatite [485]. The stability of the mineral composition of teeth also has a very long history: namely, calcium orthophosphates were found in fossil fish teeth [486]. Recent investigations of biological apatite from fossil human and animal teeth revealed its similarity to the modem biological apatite [487].

**Figure 8:**
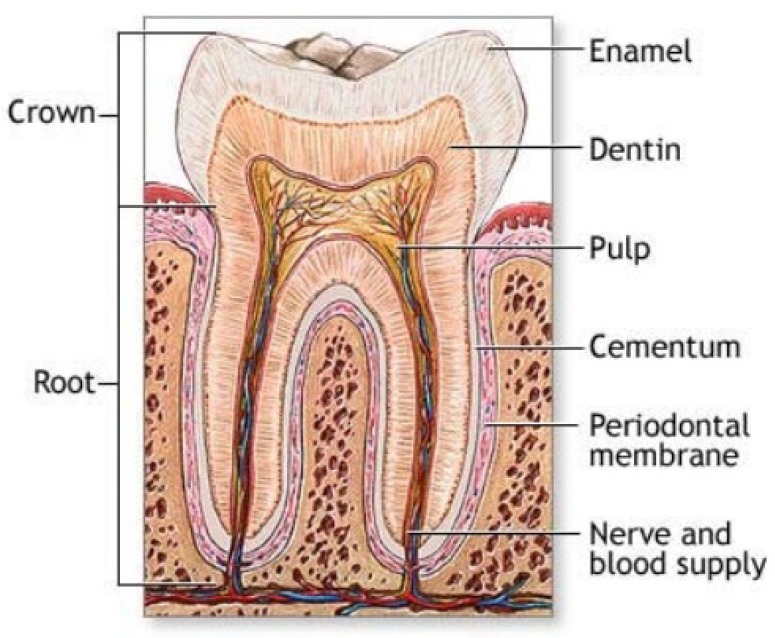
A schematic drawing of a tooth. Other very good graphical sketches of the mammalian tooth structure, including the hierarchical levels, are available in Refs. [353,400].

The structure of teeth appears to be even more complicated than that of bone (Figure 8). Unlike bone, teeth consist of at least two different materials: enamel that is a hard outer layer consisting of calcium orthophosphates and dentin, which is a bone-like inner layer, the bulk of the tooth. In addition, there is a thin layer around the tooth roots called cementum – it covers the anatomic root of the tooth. Cementum is a bone-like material similar to dentin, which connects the teeth to the jaw [488]. Finally, there is the core called pulp (commonly called “the nerve”) – it is a remnant of the embryologic organ for tooth development and contains nerves and blood vessels necessary for tooth function (Figure 8) [394,395,483,484]. Both dentin and cementum are mineralized connective tissues with an organic matrix of collagenous proteins, while the inorganic component of them consists of biological apatite. As shown in Table 1, dentin, cementum and bone are quite similar and for general purposes of material science they can be regarded as being essentially the same material [311,362,364,365,366,367,368,371,372,373,374,375,381,382,384,385,386,388,389,390,391,406,410,411,415,474,475]. Thus, most statements made in the previous chapter for bone are also valid for dentin and cementum; however, unlike bones, both dentin and cementum lack vascularization [489]. 

Dental enamel is the outermost layer of teeth. It is white and translucent and its true color can only be observed at the cutting edges of the teeth. Enamel is highly mineralized and acellular, so it is not a living tissue. Nevertheless, it is sufficiently porous for diffusion and chemical reactions to occur within its structure, particularly acidic dissolution (dental caries) and remineralization from saliva (possible healing of caries lesions). Enamel is the hardest substance in the body [387] and forms a solid, tough and wear-resistant surface for malaxation. In the mature state, it contains up to 98 % of inorganic phase (Table 1). The crystals of biological apatite of enamel are much larger as evidenced by higher crystallinity (reflecting greater crystal size and perfection) demonstrated in their X-ray diffraction patterns, than those of bone and dentin. Besides, enamel apatite has fewer ionic substitutions than bone or dentin mineral and more closely approximates the stoichiometric HA [394]. The organic phase of enamel does not contain collagen. Instead, enamel has two unique classes of proteins called amelogenins and enamelins. While the role of these proteins is not fully understood yet, it is believed that both classes of proteins aid in the enamel development by serving as a framework support [483,484,490]. The large amount of minerals in enamel accounts not only for its strength but also for its brittleness. Dentin, which is less mineralized and less brittle, compensates for enamel and is necessary as a support [483,484]. Shark enameloid is an intermediate form bridging enamel and dentin. It has enamel-like crystals of fluoridated biological apatite associated with collagen fibrils [45,309,310,311,312,313,314]. Due to the presence of fluorides, biological apatite of shark enameloid shows both higher crystal sizes and a more regular hexagonal symmetry if compared to non-fluoridated biological apatite of bones and teeth [63]. Similar correlation between the presence of fluorides and crystal dimensions was found for enamel [491].

**Figure 9:**
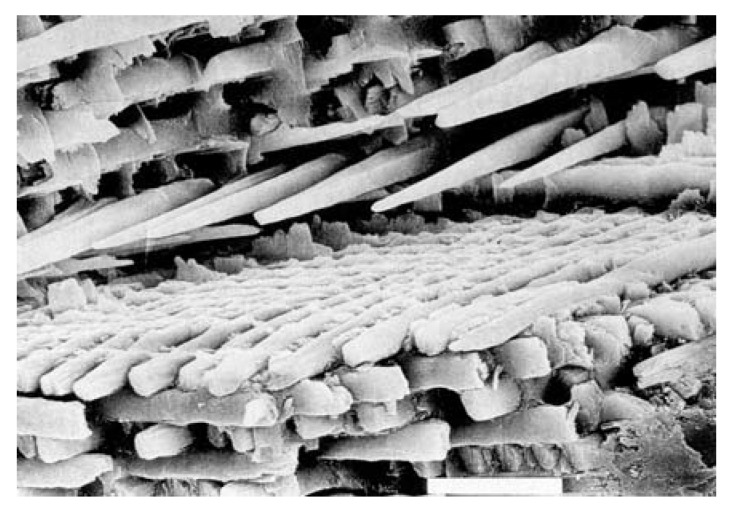
Scanning electron micrograph of the forming enamel of a continuously growing rat incisor showing ordered rods of calcium orthophosphates. Scale bar: 10 μm (taken from Ref. [311] with permission).

Like that for bones, seven levels of structural hierarchy have been also discovered in human enamel; moreover, the analysis of the enamel and bone hierarchical structure suggests similarities of the scale distribution at each level [353,401,492]. On the mesoscale level, there are three main structural components: a rod, an interrod and aprismatic enamel. Among them, the enamel rod (formerly called an enamel prism) is the basic unit of enamel. It is a tightly packed mass of biological apatite in an organized pattern. Each rod traverses uninterrupted through the thickness of enamel. They number 5 to 12 million rods per crown. The rods increase in diameter (4 up to 8 microns) as they flare outward from the dentin-enamel junction (DEJ). Needle-like enamel rods might be tens of microns long (up to 100 µm) but sometimes only 50 nm wide and 30 nm thick (Figure 9) [483,484,493,494,495,496,497,498,499,500]. They are quite different from the much smaller crystals of dentin and bone (Table 1), but all of them consist of biological apatite [296,501,502]. In cross section, an enamel rod is best compared to a keyhole, with the top, or head, oriented toward the crown of the tooth and the bottom, or tail, oriented toward the root of the tooth.

The arrangement of the crystals of biological apatite within each enamel rod is highly complex. Enamel crystals in the head of the enamel rod are oriented parallel to the long axis of the rod. When found in the tail of the enamel rod, the crystals’ orientation diverges slightly from the long axis [483,484]. The arrangement of the enamel rods is understood more clearly than their internal structure. Enamel rods are found in rows along the tooth (Figure 9) and, within each row, the long axis of the enamel rod is generally perpendicular to the underlying dentin [483,484,493,494,495,496,497]. A recent AFM study indicated that CDHA crystals in enamel exhibited regular subdomains or subunits with distinct chemical properties related to topographical features and gave rise to patterned behavior in terms of the crystal surface itself and the manner in which it responded to low pH [503].

The second structural component of the enamel matrix is the interrod (or interprismatic) enamel, which surrounds and packs between the rods. The difference between the rod and the interrod is the orientation of apatite crystals; the rod contains aligned crystallites, whereas the mineral in the interrod is less ordered. These structures coalesce to form the tough tissue of enamel, which can withstand high forces and resist damage by crack deflection. The third structure, aprismatic enamel, refers to the structures containing apatite crystals that show no mesoscale or macroscale alignment [353]. Enamel is a selectively permeable membrane, allowing water and certain ions to pass via osmosis [483,484].

The *in vivo* formation and development of teeth appears to be even more complicated when compared with the aforedescribed process of bone formation. It is a very complex biological process, by which teeth are formed from embryonic cells, grow and erupt into the mouth [396]. For human teeth enamel, dentin and cementum must all be developed during the appropriate stages of fetal development. Primary (baby) teeth start to form *in utero* between the sixth and eighth weeks, while the permanent teeth begin to form *in utero* in the twentieth week [483,484]. Recent data confirmed the necessity of calcium orthophosphates in the diet of pregnant and nursing mother to prevent early childhood dental caries [504].

As teeth consist of at least two materials with different properties (enamel and dentin), the tooth bud (sometimes called “the tooth germ” – that is an aggregation of cells that eventually forms a tooth) is organized into three parts: the enamel organ, the dental papilla and the dental follicle. The enamel organ is composed of at least four other groups of cells (for the biological details see Refs. [483,484]). Altogether, these groups of cells give rise to ameloblasts, which secrete enamel matrix proteins. The protein gel adjacent to ameloblasts is supersaturated with calcium orthophosphates, which leads to the precipitation of biological apatite. Similarly, the dental papilla contains cells that develop into odontoblasts, which are dentin-forming cells. The dental follicle gives rise to three important entities: cementoblasts, osteoblasts and fibroblasts. Cementoblasts form the cementum of a tooth. Osteoblasts give rise to the alveolar bone around the roots of teeth (see bone formation above). Fibroblasts develop the periodontal ligaments that connect teeth to the alveolar bone through cementum [394,395,396,483,484].

The first detectable crystals in enamel formation are flat thin ribbons [495,496,497], that were reported to be OCP [388,505,506,507], β-(Ca, Mg)_3_(PO_4_)_2_ [506] or DCPD [440,443]. The crystallization process of enamel is different from that for bone or dentin: amelogenin being hydrophobic self-assembles into nanospheres that guide the growth of the ribbon-like dental enamel crystals. During maturation of enamel, the mineral content increases from initially ~ 45 wt. % up to ~ 98 – 99 wt. % [440,483,484]. The enamel crystal rods widen and thicken by additional growth [440,443,508] with a simultaneous increase of the Ca/P molar ratio [508] and a decrease in carbonate content [509,510,511], finally resulting in the most highly mineralized and hardest substance produced by vertebrates. It is interesting to note that in the radular teeth of chitons, ACP was found to be the first-formed calcium orthophosphate mineral, which over a period of weeks was transformed to dahllite [512].

The crystal faces expressed in enamel are always (100) face and at the ends presumably (001) [513,514], which are the ones usually found in HA. The centers of enamel crystals contain a linear structure known as the “central dark line” (this line was also observed in bone and dentin), which consists of OCP [124,125,126,127]. As described above for bones, X-ray diffraction shows that the crystals of younger dentin are less crystalline than those of more mature dentin [474]. Therefore, maturation of dentin also means a slow transformation of biological calcium orthophosphates from ion-substituted ACP to a better-crystallized ion-substituted CDHA.

The development of individual enamel and dentin crystals was studied by high-resolution transmission electron microscopy [515,516,517]. Both processes appear to be roughly comparable and were described in a four-step process. The first two steps include the initial nucleation and formation of nanometer-sized particles of biological apatite. They are followed by ribbon-like crystal formation, which until recently was considered as the first step of biological crystal formation [515,516,517]. These complicated processes, starting with the heterogeneous nucleation of inorganic calcium orthophosphates on an organic extracellular matrix, are controlled in both tissues by the organic matrix and are under cellular control [518]. To complicate the process even further, regular and discrete domains of various charges or charge densities on the surface of CDHA crystals derived from the maturation stage of enamel development were recently discovered by a combination of atomic and chemical force microscopy [519]. Binding of organic molecules (e.g., amelogenin [519]) at physiological solution pH appears to occur on the charged surface domains of CDHA. The modern visions on dental tissue research have been reviewed recently [520].

The dentin-enamel junction (DEJ) is the interface between the dentin and enamel. It is the remnant of the onset of enamel formation because enamel grows outwards from this junction [484,521,522]. DEJ plays an important role in preventing crack propagation from enamel into dentin [523]. The major steps of enamel crystal growth at the junction have been described above but the mechanism of the junction formation is still debatable. Some authors claim that the enamel crystals grow epitaxially on the pre-existing dentin crystals because of a high continuity between enamel and dentin crystals [524,525,526]. Others have shown that enamel crystals are formed at a given distance from the dentin surface [505,506,507,527] and could either reach dentin crystals by a subsequent growth [528] or remain distant [527,529]. In addition, there are a cementum-enamel junction (CEJ) [530], who is quite similar to DEJ, and a cementum-dentin junction (CDJ) [488,531].

Enamel formation, or amelogenesis, is a highly regulated process involving precise genetic control as well as protein-protein interactions, protein-mineral interactions and interactions involving the cell membrane. Much is still unknown about the interactions between proteins present in the enamel matrix and the final crystalline phase of biological apatite [353,532]. At some point before the tooth erupts into the mouth the ameloblasts are broken down. Consequently, enamel, unlike bones, has no way to regenerate itself using the process of “active mineralization” (see aforementioned debate on bone formation) because there is no biological process that repairs degraded or damaged enamel [483,484]. In addition, certain bacteria in the mouth feed on the remains of foods, especially sugars. They produce lactic acid, which dissolves the biological apatite of the enamel in a process known as enamel demineralization that takes place below the critical pH of about 5.5. Similar process called enamel erosion occurs when a person consumes acid (citric, lactic, phosphoric, *etc*.) containing soft drinks [493,533,534,535,536]. Evidences exist that there is a preferential loss of carbonates and Mg during the acid dissolution of mineral in dental caries. Luckily, saliva gradually neutralizes the acids that cause the pH of the tooth surface to rise above the critical pH. This might cause partial enamel remineralization, the return of the dissolved calcium orthophosphates to the enamel. Until recently, it was generally agreed, that if there was sufficient time between the intake of foods (generally, two to three hours) and the damage was very limited, the teeth could repair themselves by the “passive mineralization” process [537]. Data on increased remineralization of tooth enamel by milk containing added casein phosphopeptide – ACP nanocomplexes [538] are in support of this hypothesis.

Recently, by using atomic force microscopy nano-indentation technique it was discovered that the previously demineralized samples of enamel further exposed to remineralizing solutions did show a crystalline layer of calcium orthophosphates formed on the enamel surface. Unfortunately, the re-precipitated deposits of calcium orthophosphates always consisted of loosely packed crystals and did not protect the underlying enamel from a subsequent acid attack. Furthermore, these surface deposits have been completely removed by either a toothbrush or a short exposure to an erosive acidic solution [493,539,540,541]. In this context, it should be emphasized that the term remineralization, which is often misused in the literature, should imply the process of mineral growth that goes hand in hand with a strengthening effect of the weakened enamel surface. Since no strengthening of an exposure to remineralizing solutions was observed, it might be considered that no “passive mineralization” was found (in spite of the real evidence of the re-precipitated surface deposits of calcium orthophosphates) [493,540,541]. Thus, the enamel self-repairing ability by the passive remineralization appears to be doubtful, while the active remineralization is impossible. However, investigations in this field keep going [542,543].

An amount of fluoride added to either toothpaste or mouthwash lowers the solubility of calcium orthophosphates (by formation of FHA on the surface) and therefore improves the acid-resistance of dental enamel [296,317,318,319,320,321,322,544]. Furthermore, fluorides also reduce the production of acids by bacteria in the mouth by reducing their ability to metabolize sugars.

To conclude the teeth subject, let us briefly mention on the practical application of teeth. Due to relatively small dimensions of normal teeth, only tusks and ivory of giant animals are used. For example, both the Greek and Roman civilizations used large quantities of ivory to make high value works of art, precious religious objects and decorative boxes for costly objects. Ivory was often used to form the whites of the eyes of statues. Prior to the introduction of plastics, it was used for billiard balls, piano keys, buttons and ornamental items. The examples of modern carved ivory objects are small statuary, netsukes, jewelry, flatware handles and furniture inlays.

### 4.3. Antlers

**Figure 10:**
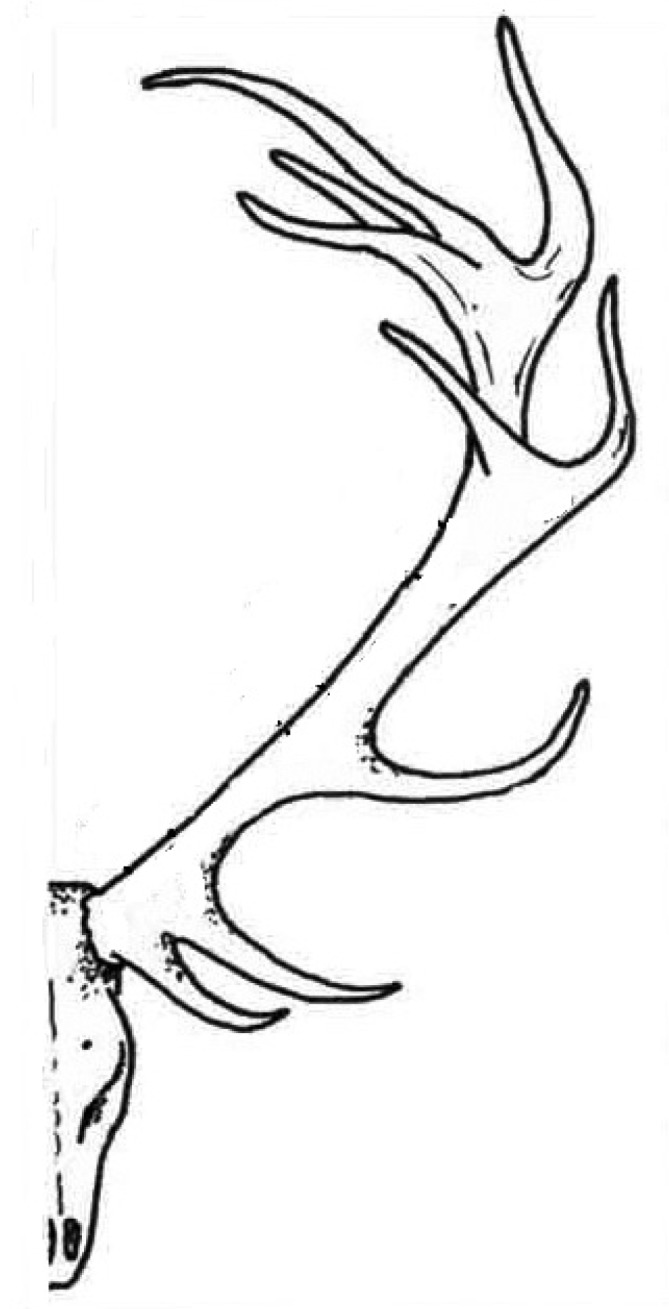
A schematic picture of a deer antler. A good cross-sectional image of a deer antler is available in Ref. [400].

Deer antlers (Figure 10) are unique biological structures since their growth rate is without parallel in vertebrates and because they are the only bony appendages in mammals capable of complete regeneration. This allows for basic research in bone biology without the interference of surgical procedures and their adverse effects in animals where samples are obtained. In addition, antlers also allow for the gathering of a large amount of samples from different populations to assess nutritional and ecological effects on bone composition and structure [545,546,547,548]. They are costly sexual secondary characters of male deer and constitute 1 to 5% of the body weight [549].

Antlers are not true horns; they are a simple extension of bone, so they have a matrix of biological apatite similar to that of mammalian bones [550]. Antlers are large and complex horn-like appendages of deer consisting of bony outgrowths from the head with no covering of keratin as is found in true horns. Usually, they begin growing in March and reach maturity in August. In winter, antlers fall off; a process known as shedding. Similar to bones, antlers contain pores and can withstand applied stresses of over 300 MPa [551,552,553,554], which is even higher than that of bones (Table 1). Therefore, antlers are occasionally considered an almost unbreakable bone [380]. Each antler grows from an attachment point on the skull called a pedicle. While an antler is growing, it is covered with highly vascularized skin called velvet, which supplies oxygen and nutrients to the growing bone. Once the antler has achieved its proper size, the velvet starts to dry out, cracks and breaks off, while the antler's bone dies. Fully developed antlers consist of dead bone only [555,556,557,558,559,560,561,562,563,564]. It was found that food processing cannot supply the mineral needs required for antler growth and thus, male deer must temporary resorb calcium orthophosphate minerals from their own skeleton for antler growth [555,556,557,558,559,560,561,562,563,564,565,566,567]. Detailed studies revealed that daily food intake provided between 25 and 40% of calcium needed for antler mineralization, which resulted in a temporary skeleton demineralization [566,567].

Antlers are a good model to study bone biology because they are accessible, shed after mating season and cast every year [568]. However, people seldom come across the antlers in the woods. Rabbits and rodents such as mice and chipmunks eat antlers (and bones of wild animals after they die) for calcium. Rodents and rabbits also gnaw bones and antlers to sharpen their incisors. Due to an extremely high growth rate, which can achieve 2 – 4 cm per day [555], combined with a very fast biomineralization, these unique appendages might be a well-suited animal model for studying the disturbances of bone formation induced by additives (*e.g.*, by excess of fluoride) [557]. Antler size and external characteristics were found to be influenced by nutrition, climatic variability and other factors. Thus, since antlers are periodically replaced, the analysis of naturally cast antlers offers the opportunity for a continuous and a noninvasive monitoring of the environmental pollution by these additives [557]. Recently, the first attempt to evaluate a potential use of deer antlers as a bone regeneration biomaterial has been performed [569]. To conclude this part, associated with aristocracy, antlers have adorned European castles and hunting lodges for centuries. Today, furnishings and accessories made from antlers are featured in fine homes throughout the world and are a reflection of grace and elegance.

## 5. Pathological Calcification of Calcium Orthophosphates

In the body of mammals, osteoblasts and odontoblasts fix ions of calcium and orthophosphate and then precipitate biological apatite onto an organic matrix. This is the process of physiological biomineralization that is restricted to the specific sites in skeletal tissues, including growth plate cartilage, bones and teeth [63,311]. Normally, mammals are supposed to die with calcium orthophosphates located in bones and teeth (and antlers for male deer) only and nowhere else, because under the normal conditions soft tissues are not mineralized. Unfortunately, owing to ageing, various diseases and under certain pathological conditions blood vessels and some internal organs are calcified as well. This process is called pathological calcification or ectopic mineralization and leads to a morbidity and a mortality [63,311,570]. In general, any type of abnormal accumulation of calcium orthophosphates in wrong places is accounted for by a disruption of systemic defense mechanism against calcification [571].

To the best of my knowledge, the first paper on a negative influence of unwanted depositions of calcium orthophosphates in the body was published as early as in 1911 [572]. This finding was confirmed in later studies [573,574]. Unwanted depositions always lead to various diseases, for instance: soft tissue calcification (in damaged joints, blood vessels, dysfunctional areas in the brain, diseased organs, scleroderma, prostate stones) [575,576,577,578,579,580], kidney and urinary stones [13,581,582,583,584], dental pulp stones and dental calculus [118,119,585,586,587], salivary stones [588], gall stones, pineal gland calcification, atherosclerotic arteries and veins [48,589,590,591,592], coronary calcification [593], cardiac skeleton, damaged cardiac valves [594], calcification on artificial heart valves [595,596,597,598,599], carpal tunnel [600], cataracts [601], malacoplakia, calcified menisci [602,603], dermatomyositis [604,605] and other [63]. In addition, there is a metastatic calcification of nonosseous viable tissue occurring throughout the body, but it primarily affects the interstitial tissue of the blood vessels, kidney, lungs and gastric mucosa [606]. A metastatic calcification is defined as a deposition of calcium orthophosphates in previously normal tissue due to an abnormal biochemistry with disturbances in the calcium or phosphorus metabolism [607]. Common causes of the metastatic calcification include hyperparathyroidism, chronic renal disease, massive bone destruction in widespread bone metastases and increased intestinal calcium absorption. One author has mentioned on “apatite diseases” which are characterized by the appearance of needle-like crystals comparable to those of bone apatite in the fibrous connective tissue [608]. All these cases are examples of a calcinosis, which might be described as a formation of calcium orthophosphate deposits in any soft tissue. In dentistry, a calculus or a tartar refers to a hardened plaque on the teeth, formed by the presence of saliva, debris and minerals. Its rough surface provides an ideal medium for bacterial growth, threatening the health of the gums and absorbing unaesthetic stains far more easily than natural teeth [13].

Contrary to the mineral phases of the normal calcifications (bone, dentine, enamel, cementum, antlers), which consist of only one type of calcium orthophosphate (namely, biological apatite), the mineral phases of abnormal and/or pathological calcifications are found to occur as single or mixed phases of other types of calcium orthophosphates (ACP, DCPD, OCP, β-(Ca,Mg)_3_(PO_4_)_2_) and/or other phosphatic and non-phosphatic compounds (*e.g.*, magnesium orthophosphates, calcium pyrophosphates, calcium oxalates, *etc.*) in addition to or in place of biological apatite (Table 4) [13,15,47,63,92,152,609,610,611,612,613]. This happens because in the places of pathological calcifications the solution pH is often relatively low. Given that nucleation and crystal growth is not a highly regulated process in any pathological deposits, there is not likely just one fundamental formation mechanism for all possible calcification types. Furthermore, various bioorganic impurities in the local environment undoubtedly influence the crystallization process, resulting in a great variety of pathological deposits. Thus, it is a highly complex problem. In some cases, the chemical composition of an unwanted inorganic phase might depend on the age of the pathological calcification and its location. For example, DCPD is more frequently found in young (3 months or younger) calculus, biological apatite is present in all ages of calculus, while β-(Ca,Mg)_3_(PO_4_)_2_ occurs more frequently in sub-gingival calculus. In mature calculus, the relative abundance of OCP, β-(Ca,Mg)_3_(PO_4_)_2_ and biological apatite also differ between the inner and outer layers [47]. It is interesting to note that the mineral phases of animal calculus (*e.g.*, from dog) was found to consist of calcium carbonate and biological apatite, while human calculi do not contain calcium carbonate [47,614].

Some findings suggested that the mechanisms and factors regulating the physiological biomineralization might be similar to those influencing the ectopic mineralization: both were initiated by various organics (*i.e.*, membrane-enclosed particles released from the plasma membrane of mineralization-competent cells), that were present [615,616,617]. In addition, other regulators (activators and inhibitors) of physiological mineralization have been identified and characterized. Besides, some evidences indicate that the same factors also contribute to the regulation of ectopic mineralization [615,616,617,618,619,620,621,622]. What’s more, the biological fluids (*e.g.*, serum, saliva, synovial fluids) are normally supersaturated with respect to biological apatite precipitation [13,47,311]; therefore, in principle, calcification is thermodynamically feasible in any part of the body. However, normally it is not the case. Therefore, in the healthy body, the appropriate inhibitory mechanisms must be at work to prevent a superfluous calcification of soft tissues. These inhibition mechanisms are a hot research topic in molecular medicine but this subject is beyond the scope of current review. The interested readers are forwarded, for example, to a very interesting review on molecular recognition at the protein/HA interface [623]. More to the point, molecular, endocrine and genetic mechanisms of arterial calcification have been reviewed in another paper [624].

**Table 4:** Occurrence of various calcium phosphates in biological systems (human) [47].

Calcium phosphate	Occurrence
biological apatite	enamel, dentin, bone, dental calculi, stones, urinary stones, soft-tissue deposits
OCP	dental calculi and urinary stones
DCPD	dental calculi, crystalluria, chrondrocalcinosis, in some carious lesions
β-(Ca, Mg)_3_(PO_4_)_2_	dental calculi, salivary stones, arthritic cartilage, soft-tissue deposits
Ca_2_P_2_O_7_·2H_2_O	pseudo-gout deposits in synovium fluids
ACP	heart calcifications in uremic patients, kidney stones

To conclude this part, it is worth remembering that calcium orthophosphates of biological origin are sparingly soluble in aqueous solutions. Removing them from the places of unwanted deposition would be an equivalent of demineralizing bone; that is a challenge. Therefore, the majority of therapeutic approaches are directed at preventing the progression of pathological calcifications. Among them, a chelation therapy might be of some interest to chemists and materials researchers because it deals with chemical processes [625,626]. Recently, the general principles of demineralization and decalcification (*i.e.*, removing the mineral Ca-containing compounds (phosphates and carbonates) from the organic matrix) have been extensively reviewed [627,628], to which the interested readers are referred.

## 6. Calcium Orthophosphates as Biomaterials and Bioceramics

A number of definitions have been developed for the term “biomaterials”. The consensus developed by experts in this field is the following: biomaterials are defined as synthetic or natural materials to be used to replace parts of a living system or to function in intimate contact with living tissue [82]. In general, biomaterials are intended to interface with biological systems to evaluate, treat, augment or replace any tissue, organ or function of the body and are now used in a number of different applications throughout the body [629,630]. *Biomaterials* are different from *biological materials* because the former are the materials that are accepted by living tissues and, therefore, might be used for tissue replacements, while the latter are the materials being produced by biological systems (wood, cotton, bones, chitin, *etc.*) [400]. In addition, there are *biomimetic materials*, which are not made by living organisms but have the composition, structure and properties similar to those of biological materials. Further, bioceramics might be defined as biomaterials of the ceramic origin. In general, it can have structural functions as joint or tissue replacements, be used as coatings to improve the biocompatibility of metal implants and function as resorbable lattices, which provide temporary structures and a framework that is dissolved and/or replaced as the body rebuilds tissue. Some types of bioceramics even feature a drug-delivery capability [631].

The performance of living tissues is the result of millions of years of evolution, while the performance of acceptable artificial substitutions those humankind has designed to repair damaged hard tissues are only a few decades old. Archaeological findings exhibited in museums showed that materials used to replace missing human bones and teeth have included animal or human (from corpses) bones and teeth, shells, corals, ivory (elephant tusk), wood, as well as some metals (gold or silver). For instance, the Etruscans learned to substitute missing teeth with bridges made from artificial teeth carved from the bones of oxen, while in the 17th century a piece of dog skull was successfully transplanted into the damaged skull of a Dutch duke. The Chinese recorded the first use of dental amalgam to repair decayed teeth in the year 659 AD, while pre-Columbian civilizations used gold sheets to heal cranial cavities following trepanation [632]. Due to the practice of cremation in many societies, not much is known about prehistoric materials used to replace bones lost to accident or disease.

In the past, many implantations failed because of infection or a lack of knowledge about toxicity of the selected materials. In this frame, the use of calcium orthophosphates as biomaterials and bioceramics is based upon their similarity with the mineral phase of bone and teeth [311,312,362,363]. However, according to available literature, the first attempt to use calcium orthophosphates (it was TCP) as an artificial material to repair surgically created defects in rabbits was performed in 1920 [633]. In general, calcium orthophosphate-based biomaterials can be prepared from various sources [634,635]. Unfortunately, up to now, all attempts to synthesize bone replacement materials for clinical applications featuring the physiological tolerance, biocompatibility and a long term stability have had only a relative success; it comes to show a superiority and a complexity of the natural structures [355].

Generally, living organisms might treat artificial implants as bioinert, biotolerant, bioactive or bioresorbable biomaterials [82,629,630,636]. Bioinert (*e.g.*, zirconia, alumina, carbon and titanium) and biotolerant (*e.g.*, polymethyl methacrylate (PMMA), titanium and Co-Cr alloy) materials will evoke a physiological response to form a fibrous capsule, thus, isolating the material from the body. Calcium orthophosphates (both non-substituted and ion-substituted) fall into the categories of bioactive and bioresorbable materials [636]. A bioactive material will dissolve slightly but promote the formation of a layer of biological apatite before interfacing directly with the tissue at the atomic level, that result in the formation of a direct chemical bond with bone. Such an implant will provide a good stabilization for materials that are subject to mechanical loading. A bioresorbable material will dissolve and allow a newly formed tissue to grow into any surface irregularities but may not necessarily interface directly with the material [289,637,638,639,640,641]. Bioceramics made of dense HA would be a good example of a bioactive material, while porous scaffolds made of BCP (*i.e.,* β-TCP + HA [151,152,153,154,155,156,157,158,159,160], α-TCP + HA [163,164,165,166]) or bone grafts made of CDHA [642], TCP [643] and/or ACP [199,644] appear to be the examples of bioresorbable materials. Unfortunately, calcium orthophosphate bioceramics possess poor mechanical properties that do not allow them to be used in load-bearing areas [645]. For this reason, the medical applications of calcium orthophosphates are currently focused on the production of non-load-bearing implants, such as pieces for middle ear surgery, filling of bone defects in oral or orthopedic surgery, as well as coating of dental implants and metallic prosthesis [355,646]. The mechanical properties of calcium orthophosphate biomaterials and bioceramics have been reviewed elsewhere [266,647]. In addition, there is a good review on the recent developments in processing and surface modification of HA [648].

In spite of serious mechanical limitations, biomaterials and bioceramics of calcium orthophosphates are available in various physical forms: particles, blocks (dense or porous), injectable compositions, self-setting cements, coatings on metal implants, composites with polymers, *etc.* [649]. A porous surface provides mechanical fixation in addition to providing sites on the surface that allow chemical bonding between biomaterials and bone [650]. For example, porous HA bioceramics can be colonized by bone tissues [651,652,653]. Therefore, macroporosity (pore size > 100 μm) in solid biomaterials is intentionally introduced by addition of various porogens, which consist of crystals or particles of either volatile or soluble substances (*e.g.*, naphthalene, sucrose, NaHCO_3_, gelatin, PMMA microbeads) [152,289,654,655,656,657,658]. Sintering particles, preferably spheres of equal size, is another way to generate porous three-dimensional (3D) bioceramics of calcium orthophosphates. A wetting solution such as polyvinyl alcohol is usually used to aid compaction, which is achieved by cold isostatic pressing the particles into cylinders at approximately 200 MPa [659]. As hardly any effect of macropore sizes (150, 260, 510 and 1220 μm) on the *in vivo* response was observed [660], there is no need to create bioceramics with very big pores; however, the pores must be interconnected. Microporosity (pore size < 10 μm) results from the sintering process, while dimensions of the pores depend on temperature and sintering time. Creation of the desired porosity in bioceramics is a rather complicated engineering task and the interested readers are referred to the special literature [152,199,656,661,662,663,664,665,666,667,668,669,670,671,672].

The sintering stage appears to be of a great importance to produce bioceramics with the required properties. Several processes occur during sintering of calcium orthophosphates. Firstly, moisture, carbonates and all volatile chemicals remaining from the synthesis stage, such as ammonia, nitrates and any organic compounds, are removed as gaseous products. Secondly, the removal of these gases facilitates the production of dense materials during sintering. Thirdly, these chemical changes are accompanied by a concurrent increase in crystal size and a decrease in the specific surface area. Fourthly, there is the chemical decomposition of all acidic orthophosphates and their transformation into other phosphates (*e.g.,* 2HPO_4_^2-^ → P_2_O_7_^4-^ + H_2_O). Besides, sintering causes toughening of the ceramics [673]. Further details on the sintering processes of calcium orthophosphates are available elsewhere [13,14,233,266,289,641].

Studies showed that increasing the specific surface area and pore volume of biomaterials for tissue repair might greatly accelerate the kinetic process of biological apatite deposition and therefore enhance the bone-forming bioactivity [674]. More importantly, the precise control over porosity, pore size and internal pore architecture of biomaterials on different length scales is essential for understanding of the structure-bioactivity relationship and the rational design of better bone-forming biomaterials [675,676].

Calcium orthophosphates in a number of forms and compositions are currently either in use or under consideration in many areas of dentistry and orthopedics, with even more in development. For example, bulk material, available in dense and porous forms, is used for alveolar ridge augmentation, immediate tooth replacement and maxillofacial reconstruction [13,46,677]. Further applications include orbital implants (Bio-Eye^®^) [678,679], increment of the hearing ossicles, spine fusion and repair of bone defects [680,681]. In order to permit growth of new bone into bone defects, a suitable bioresorbable material should fill the defects. Otherwise, ingrowth of fibrous tissue might prevent bone formation within the defects. Today, a large number of different calcium orthophosphate bioceramics for the treatment of bone defects is available on the market. As an example, the readers are referred to a thorough physicochemical characterization of 14 calcium phosphate-based bone substitution materials in comparison to natural bone [682]. Commercial and trade names of several very important types of calcium orthophosphate bioceramics may be found in the iterature [682,683].

**Figure 11:**
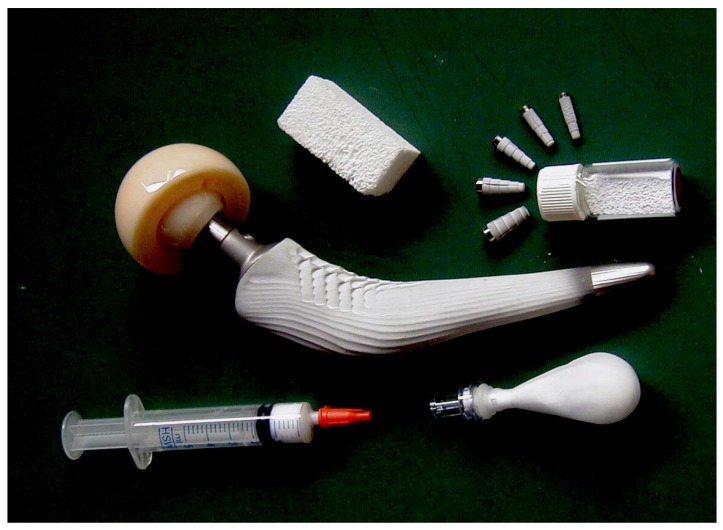
General appearance of various commercial calcium orthophosphate-based bone graft materials.

Chemically, calcium orthophosphate bioceramics are based on HA, β-TCP, α-TCP and/or BCP (*i.e.*, a composite of HA with α- or β-TCP) [13,46,151,152,153,154,155,156,157,158,159,160,161,164,165,166,167,169,172,173,677,684]. The BCP concept is determined by the optimum balance of a more stable phase of HA and a more soluble TCP. General requirements for the ideal bone grafts are as follows: pores of some 100 µm size, a biodegradation rate comparable to the formation of bone tissue (*i.e.*, between a few months and about two years) and the sufficient mechanical stability. When compared to α- and β-TCP, HA is a more stable phase under the physiological conditions, as it has a lower solubility and a slower resorption kinetics [13,46,677]. As implants made of calcined HA are present in bone defects for many years after implantation, bioceramics made of β-TCP, α-TCP, CDHA or BCP [63,153,155,156,163,164,165,166,167,168,169,172,173,685] are more preferable for medical purposes. According to both observed and measured bone formation parameters, calcium orthophosphates were ranked as follows: low sintering temperature BCP (rough and smooth) ≈ medium sintering temperature BCP ≈ TCP > calcined low sintering temperature HA > non-calcined low sintering temperature HA > high sintering temperature BCP (rough and smooth) > high sintering temperature HA (calcined and non-calcined) [685]. In the case of BCP, its biodegradation kinetics depends on the HA/β-TCP ratio: the higher the ratio, the lower the degradation rate [157]. Figure 11 shows some randomly chosen examples of commercially available calcium orthophosphate bioceramics for use as bone grafts.

The need of biomaterials for minimal invasive surgery has induced the development of a concept of self-setting bone cements made from calcium orthophosphates to be applied as injectable and/or mouldable bone substitutes [78,84,85,86,87,93,94,95,96,106,107,108,109,110,144,145,146,147,170,171,338,654,655,668,685,686,687,688,689]. This is a low temperature bioceramics. Two major types of cements are possible. The first one is a dry mixture of two different calcium orthophosphates (a basic one and an acidic one), in which, after being wetted, the setting reaction occurs according to an acid-base reaction. The second type of calcium orthophosphate cements is when the initial and final calcium orthophosphates have the same Ca/P molar ratio. Typical examples are ACP with Ca/P molar ratio within 1.50 – 1.67 and α-TCP: they form CDHA upon contact with an aqueous solution [685,686]. Upon mixing with water, initial calcium orthophosphate(s) are dissolved and precipitated into less soluble calcium orthophosphates, which causes the cement setting. During the precipitation reaction, new crystals grow and become entangled, thus providing a mechanical rigidity to the cement. Setting of these cements occurs mostly within the initial 6 hours, yielding a ~ 80% conversion to the final products and a compressive strength of 40 – 60 MPa. The rate of hardening is strongly influenced by a powder to liquid ratio and addition of other chemicals [106,170,685,686,687,688,689]. Despite a large number of formulations, all calcium orthophosphate cements can only form two different end products: CDHA and DCPD [685,686].

The first animal study on a calcium orthophosphate cement was performed in 1991: the cement consisting of TTCP and DCPA was investigated histologically by implanting disks made of this cement within the heads of nine cats [690,691]. All calcium orthophosphate cements are biocompartible, bioactive and bioresorbable. The structure and composition of the hardened cements is close to that of bone mineral; therefore, the material of these cements can easily be used by bone remodeling cells for reconstruction of damaged parts of bones [170,685,686]. The biomechanical evaluation of calcium orthophosphate cements for use in vertebroplasty might be found elsewhere [692]. Unfortunately, the cements possess a low mechanical strength; this property might be improved by reinforcement with polymers [693]. A good adaptation to the defect geometry is the major advantage of bone cements, when compared to implantation of bulk ceramics and scaffolds [78,170,266,338,685,686,689].

Injectable bone substitutes (IBS) made of calcium orthophosphates and an aqueous solution of a hydrophilic biodegradable polymer are another type of biomaterials for minimal invasive surgery [156,694,695,696,697,698,699,700]. They look as pastes of a high viscosity but possessing enough fluidity to be injected into bone defects by a standard syringe with a needle. These materials are perfectly biocompatible and potentially resorbable and, thanks to their initial plasticity, they can fit bone defects very easily, without necessity to elaborate shaping of implantation site. During bioresorption of IBS, bone cells are able to invade the spaces released by disappearance of the biodegradable polymer. Creation of the required level of viscosity to prevent IBS from segregation and phase separation during the shelf life is the major task of the polymer in IBS, while calcium orthophosphates is the building material for bone healing. In terms of application, IBS more or less similar to the aforementioned bone cements but, unlike the cements, IBS do not possess the self-setting abilities since no chemical reactions occur between the components [701]. Besides, there are paste-like formulations consisting of a suspension of pure HA in water prepared by a wet chemical reaction [702,703,704]. Further details on IBS are available elsewhere [156,694,695,696,697,698,699,700]. Recently, injectable and macroporous calcium orthophosphate cement scaffolds, combining the advantages of IBS and bone cements, have been developed [705]. The future development of both IBS and calcium orthophosphate bone cements can be seen in introduction of living cells into their composition [706,707].

Calcium orthophosphate coatings on metals are often applied in medicine [288]. Metallic implants are encountered in endoprostheses (total hip joint replacements) and artificial teeth sockets. The requirement for a sufficient mechanical stability necessitates the use of a metallic body for such devices. As metals usually do not undergo bone bonding, *i.e.* do not form a mechanically stable link between the implant and bone tissue, ways have been sought to improve the mechanical contact at the interface [708,709]. The major way is to coat the metal with calcium orthophosphate ceramics that generally exhibit bone-bonding ability between the metal and bone [636,710,711]. The list of most important coating techniques is comprised in Table 5, while the main advantages and drawbacks of each coating technique, as well as the important properties of the deposed calcium orthophosphates, are discussed in details elsewhere [268,288,289,712,713,714,715,716,717,718,719,720]. Clinical results for HA-coated metallic implants revealed that they had much longer life times after implantation than uncoated devices. Namely, HA coating as a system of fixation of hip implants was found to work well in the short to medium term (8 years [721], 15 years [722], 17 years [723] and 19 years [724]). Similar data for HA-coated dental implants are also available [725,726]. The longer-term clinical results are awaited with a great interest. The biomedical aspects of osteoconductive [727] coatings for total joint arthroplasty have been reviewed elsewhere [728].

The perfect biomaterial for medical applications would not only be biocompatible but also have physical properties similar to those of the tissue being replaced or repaired. Researchers therefore have sought ways of combining calcium orthophosphate bioceramics with other materials to tailor properties such as strength and elasticity to meet system requirements. This has led to a large variety of bone substituting composites and hybrid biomaterials made of calcium orthophosphate bioceramics and (bio)organic compounds (usually, polymers, preferably, biodegradable ones). This approach appeared due to the poor mechanical properties (namely: a low elasticity, a high brittleness, a low tensile strength, a low fracture toughness and a poor impact resistance) of bone substitutes made of calcium orthophosphates only [266,271,272]. In addition, it is worth reminding that all biologically formed calcified tissues (bones, teeth, antlers, shells, *etc*.) appear to be very complicated composites of (bio)organic and inorganic phases [13,46,47,63,258,311,362,363]. In such composites, the mineral component provides the strength whereas the (bio)organic component contributes to the ductility. This combination of strength and ductility leads to an energy absorption prior to failure [390]. A list of the suitable calcium orthophosphates (except of MCPM and MCPA – both are too acidic and, therefore, are not biocompatible) is mentioned in Table 2, while there is an even greater choice of biocompatible polymers those can be divided into two major groups: synthetic polymers (*e.g.*, polyesters, PMMA, poly-ε-caprolactone) and polymers of biological origin (*e.g.*, collagen, gelatin, chitosan, alginate, modified starch, cellulose esters). Various ways have been already realized to bring these two components together into biocomposites, like simple mechanical mixing or co-precipitation. Usually, powder forms of calcium orthophosphates are used to produce biocomposites. It is also possible to introduce porosity into such biocomposites that is advantageous for most applications as bone substitution material. Such biocomposites might possess the unique properties; for example, there is a recent report on shape memory properties of poly(D,L-lactide)/HA biocomposites [729].

The topic of the composite biomaterials made of calcium orthophosphates and organic/biological compounds was first introduced in 1981 by Prof. William Bonfield, who realized the application potential of calcium orthophosphates as fillers in polymer-bioceramics biocomposites and the move was envisaged towards an improved mechanical performance of HA bioceramics [730]. Biocomposites of polymers and calcium orthophosphate bioceramics can confer favorable mechanical properties, including strength due to the ceramic phase, toughness and plasticity due to the polymer phase, as well as a graded mechanical stiffness. Another advantage of such biomaterials is that they are sufficiently soft and ductile to be shaped by a surgeon in the operating theatre. Although current technologies can yet reproduce neither the multistage biosynthesis, nor the hierarchical structure, nor the mechanical properties of bones, the synthesis of various types of calcium orthophosphate-based biocomposites and hybrid biomaterials is a strong and very promising research area. The interested readers are referred to other papers and reviews on this subject [289,390,391,731,732,733,734,735,736,737,738,739,740,741,742,743].

To conclude this part, the bioactivity mechanism of calcium orthophosphate biomaterials and bioceramics should be described. Strangely enough, a careful search in the literature revealed only three publications [268,744,745] where the bioactivity mechanism of calcium orthophosphates had been briefly described. For example, the chemical changes occurring after exposure of a synthetic HA ceramic to both *in vivo* (implantation in human) and *in vitro* (cell culture) conditions were studied. A small amount of HA was phagocytized but the major remaining part behaved as a secondary nucleator as evidenced by the appearance of a newly formed mineral [744]. *In vivo*, the cellular activity (*e.g*., by macrophages or osteoclasts) associated with acidic environment were found to result in partial dissolution of calcium orthophosphate bioceramics, causing liberation of calcium and orthophosphate ions onto the microenvironment. The liberated ions increased the supersaturation condition of the biologic fluid, causing precipitation of biological apatite nanocrystals with simultaneous incorporating of various ions presented in the biologic fluid. Infrared spectroscopic analyses demonstrated that these nanocrystals were intimately associated with an organic component (probably proteins) that might also have originated from the biologic fluid or serum [745].

**Figure 12:**
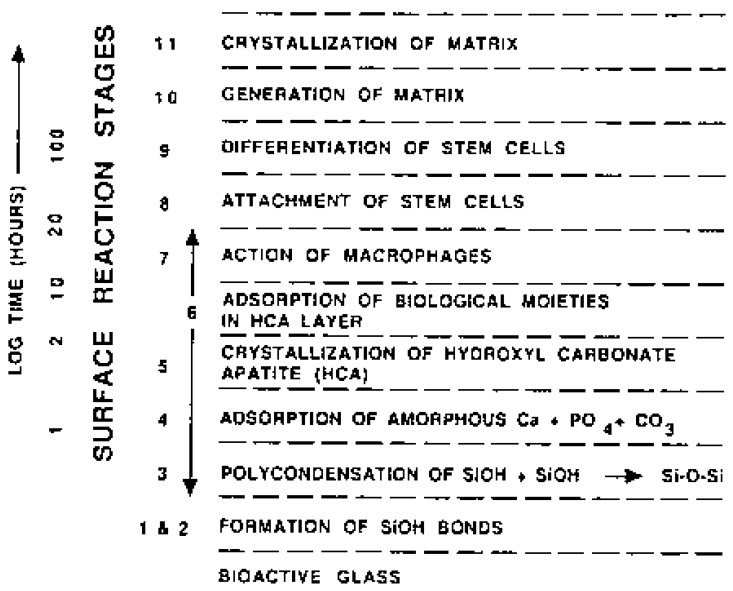
The sequence of interfacial reactions involved in forming a bond between tissue and bioactive glasses. The border between “dead” and “alive” occurs approximately at stage 6. Reprinted from Ref. [272] with permission.

Therefore, one should better rely on the bioactivity mechanism of other biomaterials, particularly of bioactive glasses – the concept introduced by Hench [271,272]. The mechanism of bonding of bioactive glasses to living tissue involves a sequence of 11 successive reaction steps. The initial five steps occurred on the surface of biomaterials are “chemistry” only, while the remaining six steps belong to “biology” because the latter include colonization by osteoblasts, followed by proliferation and differentiation of the cells to form a new bone that had a mechanically strong bond to the implant surface (Figure 12). Therefore, in the case of bioactive glasses the border between “dead” and “alive” is located between stages 5 and 6.

**Table 5:** Various techniques to deposit bioresorbable coatings of calcium orthophosphates on metal implants [268,712].

Technique	Thickness	Advantages	Disadvantages
Thermal spraying	30 – 200 μm	High deposition rates; low cost	Line of sight technique; high temperatures induce decomposition; rapid cooling produces amorphous coatings
Sputter coating	0.5 – 3 μm	Uniform coating thickness on flat substrates; dense coating	Line of sight technique; expensive; time consuming; produces amorphous coatings
Pulsed laser deposition	0.05-5 μm	Coating by crystalline and amorphous phases; dense and porous coating	Line of sight technique
Dynamic mixing method	0.05-1.3 μm	High adhesive strength	Line of sight technique; expensive; produces amorphous coatings
Dip coating	0.05 – 0.5 mm	Inexpensive; coatings applied quickly; can coat complex substrates	Requires high sintering temperatures; thermal expansion mismatch
Sol-gel technique	< 1 μm	Can coat complex shapes; low processing temperatures; relatively cheap as coatings are very thin	Some processes require controlled atmosphere processing; expensive raw materials
Electrophoretic deposition	0.1 – 2.0 mm	Uniform coating thickness; rapid deposition rates; can coat complex substrates	Difficult to produce crack-free coatings; requires high sintering temperatures
Biomimetic coating	< 30 μm	Low processing temperatures; can form bonelike apatite; can coat complex shapes; can incorporate bone growth stimulating factors	Time consuming; requires replenishment and a pH constancy of simulated body fluid
Hot isostatic pressing	0.2 – 2.0 μm	Produces dense coatings	Cannot coat complex substrates; high temperature required; thermal expansion mismatch; elastic property differences; expensive; removal/interaction of encapsulation material
Electrochemical deposition	0.05 – 0.5 mm	Uniform coating thickness; rapid deposition rates; can coat complex substrates; moderate temperature, low cost	The coating/substrate bonding is not strong enough

According to Hench, all bioactive materials “form a bone-like apatite layer on their surfaces in the living body and bond to bone through this apatite layer. The formation of bone-like apatite on artificial material is induced by functional groups, such as Si – OH (in the case of biological glasses), Ti – OH, Zr – OH, Nb – OH, Ta – OH, – COOH and – H_2_PO_4_ (in the case of other materials). These groups have specific structures revealing negatively charge and induce apatite formation via formations of an amorphous calcium compound, *e.g.*, calcium silicate, calcium titanate and ACP” [271,272]. For want of anything better, the bioactivity mechanism of calcium orthophosphates can also be described by Figure 12 by omission of several initial stages, as it was actually made for HA in Refs. [268,745], where three initial chemical stages of the Hench’s mechanism were replaced by partial dissolution of HA.

## 7. Biomimetic Crystallization of Calcium Orthophosphates

In general, biomimetics [746] (also known as bionics, biognosis and/or biomimicry) might be defined as application of the methods and systems found in nature to the study, design and construction of new engineering systems, materials, chemical compounds and modern technology. The concept is very old (*e.g.*, the Chinese wanted to make artificial silk ~ 3,000 years ago; Daedalus’ wings were one of the early design failures) but the implementation is gathering momentum only recently. In spite of the tremendous achievements of modern science and technology, the nature’s ability to assemble inorganic compounds into hard tissues (shells, spicules, teeth, bones, antlers, skeletons, *etc.*) is still not achievable by the synthetic procedures. This is not surprising – designs found in nature are the result of millions of years of evolution and competition for survival. The models that failed are fossils; those that survived are the success [747]. In the frames of this review, biomimetics is considered as mimicking natural manufacturing methods to generate artificial calcified tissues (grafts, implants, prostheses) those might be used as temporary or permanent replacements of the missing, lost, injured or damaged bones and teeth. It is important to notice, that precipitation of calcium orthophosphates and calcium carbonates have been considered to correlate with bone formation, at least, since 1923 [748].

As this is mainly the subject of crystallization of calcium orthophosphates, the matter of choosing the correct experimental conditions and well-mimicking solutions is of the primary importance. The easiest way to perform the crystallization would be mixing of aqueous solutions containing the ions of calcium and orthophosphate [13,14,15]. Unfortunately, such type of crystallization provides precipitates with the properties (chemical composition, Ca/P ratio, crystallinity level, particle size distribution, *etc*.) far different from those of biological apatite. This can be explained by the following paramount differences between the *in vivo* and *in vitro* crystallization conditions [749]:

(i) *In vitro* crystallization normally occurs at permanently depleting concentrations of calcium and orthophosphate, while the concentrations of all ions and molecules are kept strictly constant during biological mineralization (the same is valid for the solution pH);
(ii) Chemical crystallization is a fast process (time scale of minutes to days), while the biological process is a slow one (time scale of weeks to years);
(iii) Many inorganic, bioorganic, biological and polymeric compounds are present in biological liquids (blood plasma, serum, saliva). Each of these compounds might act as an inhibitor, promoter, nucleator or even as a template for the growth of biological apatite [355]. In addition, each of them somehow influences the crystallization kinetics and might be either incorporated into the solid structure or co-precipitated with calcium orthophosphates.
(iv) Chemical crystallization is, by all means, a “passive” process, while the biological mineralization is strongly influenced by cells and occurs by the self-organization mechanisms [396,419,420]. Still there are no good ways to overcome this difference.

The first and the second differences might be overcome by using the appropriate crystallization techniques. The details are available elsewhere [749] but, briefly, the first problem can be overcome by either a continuous flow of a supersaturated solution [750,751] or using a constant-composition (CC) technique [129,752,753]. The second difference can be surpassed by a restrained diffusion of calcium and orthophosphate ions from the opposite directions in, for example, a double-diffusion (DD) crystallization device or in viscous gels [294,295,296,298,299,754]. The CC and DD techniques have been combined into a single constant-composition double-diffusion (CCDD) device, which currently seems to be the most advanced experimental tool to perform biomimetic crystallization [749,755,756,757,758,759]. However, in no case the CCDD device should be considered as the final construction; it still has much room for further improvement, *e.g.* by upgrading the design of the crystallization chamber [760]. Other constructions, *e.g.* to study calcification of biological heart valve prostheses [761], are also possible. In addition, one should keep in mind that the potential of the standard CC technique has not reached its limit yet: for example, recently a good mimicking of the self-organized microstructure of tooth enamel has been achieved [762].

The third major difference between the *in vivo* and *in vitro* crystallization conditions could be overcome by using the appropriate crystallization solutions [749]. The presence of calcium and orthophosphate ions in some biological fluids has been known, at least, since 1921 [763,764]. Therefore, the best way would be to perform experiments using natural liquids (blood serum, saliva, lymph, *etc*.), but this is not easy due to variability of the chemical and biochemical composition of natural liquids and problems with their storage. As stated before, using supersaturated aqueous solutions containing only the ions of calcium and orthophosphate appears to be unable to mimic the crystallization of biological apatite; therefore, more advanced solutions have been elaborated. To the best of my knowledge, Hanks’ balanced salt solution (HBSS) [765] was the first successful simulating medium, containing the ions of calcium and orthophosphate together with other inorganic ions and glucose. HBSS is commercially available and still used in biomimetic experiments [766,767,768]; its chemical composition might be taken, *e.g.*, from Refs. [769,770]. Other popular physiological solutions include α-modified Eagle’s [771] medium (α-MEM) and its variation Dulbecco’s [772] modified Eagle’s medium (DMEM), which contain numerous bioorganic (*e.g.*, alanine, aspartic acid, glycine, biotin, vitamin C, folic acid, riboflavin) and inorganic (*e.g.*, CaCl_2_, KCl, NaCl, NaH_2_PO_4_) components [773,774,775], phosphate buffered saline (PBS) that contains only inorganic components (*e.g.*, CaCl_2_, MgCl_2_, KCl, KH_2_PO_4_, NaCl, NaH_2_PO_4_) [776,777], as well as an artificial saliva containing both bioorganic (*e.g.*, xantan gum or sodium carboxymethylcellulose, sorbitol, *etc*.) and inorganic (*e.g.*, CaCl_2_, MgCl_2_, KCl, KH_2_PO_4_, NaCl, KH_2_PO_4_) compounds [778,779]. All these simulating solutions are commercially available.

However, the most popular biomimetic solution is a protein-free acellular simulated body fluid (SBF). It was introduced by Kokubo *et al.* [780] and occasionally named as Kokubo’s SBF. It is a metastable aqueous solution with pH ~ 7.40, supersaturated with respect to the precipitation of OCP, β-TCP, CDHA and HA [781], containing only inorganic ions in concentrations nearly equal to those in human blood plasma. However, the standard SBF formulation, firstly, contains the tris/HCl buffer, and, secondly, the concentration of hydrogencarbonate (4.2 mM) is only a fraction of that in blood plasma (27 mM) [780]. The problem of a low concentration of hydrogencarbonate ions has been overcome by first introducing a “synthetic body fluid” [782,783,784] and later a revised SBF (rSBF) [785,786]. Due to the chemical similarity with human blood plasma, rSBF currently seems to be the best simulating solution. However, it contains Hepes buffer, loses CO_2_ in open vessels and does not contain any organic and/or biological molecules [785,786]. Other types of SBF are also available; the interested readers are referred to a leading opinion co-authored by the inventor of SBF [787], where the entire history and the classical preparation techniques of various SBF formulations are well described. Recently, another leading opinion on the suitability of SBFs for the *in vitro* bioactivity tests was published [788]. The authors demonstrated that (i) there is presently no enough scientific data to support the SBF suitability and (ii) even though bioactivity tests with SBFs are valid, the way the tests are generally conducted leaves room for further improvements. Furthermore, the preparation protocol of SBF solutions was reconsidered and a new procedure was suggested to improve the reproducibility of bioactivity tests [788]. The application of SBF for the surface mineralization of various materials *in vitro* has been reviewed in Ref. [789], while the theoretical analysis of calcium orthophosphate precipitation (the driving force and the nucleation rate based on the classical crystallization theory) in SBF is also available [781].

Further attempts to improve the biomimetic properties of SBF and rSBF have been performed [787,788]. Efforts were made to replace artificial buffers (tris/HCl, Hepes) with simultaneous increasing the concentration of hydrogencarbonates for SBF [790,791] or avoiding losses of CO_2_ from open vessels for rSBF [749,755,756,757,758,759] by means of permanent bubbling of gaseous CO_2_ through the solutions. Addition of the most important organic and biological compounds like glucose [757] and albumin [755] is another direction to improve biomimetic properties of SBFs; further improvements of biomimetic solutions are to be made in future. Occasionally, condensed solutions of SBF (*e.g.,* 1.5-fold, 2-fold [792,793], 5-fold [794,795] and even 10-fold [796]) are used to accelerate the precipitation; however, whenever possible this should be avoided because the application of condensed solutions of SBF leads to changes in the chemical composition of the precipitates; namely, the concentration of carbonates increases, while the concentration of orthophosphates decreases [797].

It is very difficult to mimic exactly the calcification process that occurs in bones and teeth. A step further would be to perform the precipitation from the simulating solutions on templates of biomineralization proteins for the control of crystal organization and properties. For example, there are successful attempts to crystallize calcium orthophosphates on collagen in order to obtain bone-like composites [391,798,799,800,801,802,803]. Such collagen/calcium orthophosphate composites are currently under investigation for clinical use. Other popular biomimetic matrixes to perform calcium orthophosphate crystallization comprise gelatin [294,295,296,298,299,804,805,806], chitosan [804,807,808], organic polyelectrolytes [809,810,811,812], titanium and its alloys [813,814,815,816,817,818,819], polymers [820], cellulose [821], self-assembled monolayers [822] and many other materials. Some of such materials are occasionally called “organoapatites” [353].

## 8. Calcium Orthophosphates in Tissue Engineering

All present day orthopedic implants lack three of the most critical characteristics of living tissues: (i) the ability to self-repair; (ii) the ability to maintain a blood supply; (iii) the ability to modify their structure and properties in response to environmental factors such as mechanical load [667]. Needless to say, bones not only possess all the aforementioned properties but, in addition, they are self-generating, hierarchical, multifunctional, nonlinear, composite and biodegradable; therefore, good artificial bone substitutes must possess similar properties [355]. 

The last two decades have seen a surge in creative ideas and technologies developed to tackle the problem of repairing or replacing diseased and damaged tissues, leading to the emergence of a new field in healthcare technology now referred to as *tissue engineering*. Tissue engineering is an interdisciplinary field that exploits a combination of living cells, engineering materials and suitable biochemical factors in a variety of ways to improve, replace, restore, maintain or enhance living tissues and whole organs [823]. This field of science [824] started more than a decade ago [825] and nowadays is at full research potential due to the following key advantages: (i) the solutions it provides are long-term, much safer than other options and cost-effective as well; (ii) the need for a donor tissue is minimal, which eliminates the immuno-suppression problems; (iii) the presence of residual foreign material is eliminated as well. As two of three major components (namely, living cells and biochemical factors) of tissue engineering appear to be far beyond the scope of this review, here the topic of tissue engineering is narrowed down to the engineering materials only.

Cells are generally implanted or seeded into an artificial structure, usually referred to as a scaffold, capable of supporting 3D tissue formation. The scaffolds are temporary matrices for bone growth and provide a specific environment and architecture for tissue development. They serve at least one of the following purposes: (i) allow cell attachment and migration; (ii) deliver and retain cells and biochemical factors; (iii) enable diffusion of vital cell nutrients and expressed products; (iv) exert certain mechanical and biological influences to modify the behavior of the cell phase [826]. To achieve the goal of tissue reconstruction, the scaffolds must meet some specific requirements. A reasonable surface roughness is necessary to facilitate cell seeding and fixation [827,828,829]. A high porosity and an adequate pore size are very important to provide diffusion throughout the whole structure of both cells and nutrients [828,829,830,831,832,833,834]. Biodegradability is very essential since scaffolds need to be resorbed by the surrounding tissues without the necessity of a surgical removal. The resorption rate has to coincide as much as possible with the rate of tissue formation [835]: this means that while cells are fabricating their own natural matrix structure around themselves, the scaffold is able to provide a structural integrity within the body and eventually it will break down leaving the newly formed tissue that will take over the mechanical load. Injectability is also an important factor for the clinical applications [685,686,694,695,696,697,698,699].

In the case of bone grafts, the aim of tissue engineering is to provide an artificially prepared porous scaffold made of calcium orthophosphates that provides the physical and chemical cues to guide cell seeding, differentiation and assembly into 3D tissues of a newly formed bone [836,837,838,839,840]. More to the point, bone-forming functions of cells can be dependent on grain morphology of the scaffolds. For example, osteoblast functions were found to be increased on nanofiber structures if compared to nanospherical ones because nanofibers more closely approximated the shape of biological apatite in bones [841]. To meet these needs, much attention is devoted to further modification of calcium orthophosphates [842]. From the chemical point of view, the modifications include synthesis of novel ion-substituted calcium orthophosphates [843,844,845,846,847,848], while from the material point of view the major research topics include nanocrystalline structures [290,849,850,851,852,853,854,855,856,857,858], organic-inorganic composites and hybrid biomaterials [289,731,732,733,734,735,736,737,738,739,858,859,860], fibers and whiskers [861,862,863,864,865,866,867,868], micro- and nanospheres [859,869,870,871,872], porous 3D scaffolds made of ACP [199], HA [873,874,875,876] and BCP [877], structures with graded porosity [878] and hierarchically organized ones [879]. The influence of the porosity of HA ceramics on *in vitro* and *in vivo* bone formation studied by cultured rat bone marrow stromal cells has been studied [880]. The feasible production of ceramic scaffolds with tailored structure and properties opens up a spectacular future for calcium orthophosphates.

There are three principal therapeutic strategies for treating diseased or injured tissues in patients: (i) implantation of freshly isolated or cultured cells; (ii) implantation of tissues assembled *in vitro* from cells and scaffolds; (iii) *in situ* tissue regeneration. For cellular implantation, individual cells or small cellular aggregates from the patient or a donor are either injected into the damaged tissue directly or are combined with a degradable scaffold *in vitro* and then implanted. For tissue implantation, a complete 3D tissue is grown *in vitro* using patient or donor cells and a bioresorbable scaffold and then is implanted into the patients to replace diseased or damaged tissues. For *in situ* regeneration, a scaffold implanted directly into the injured tissue stimulates the body’s own cells to promote local tissue repair [823,881]. In any case, simply trapping cells at a particular point on a surface is not enough: the cells must be encouraged to differentiate, which is impossible without the presence of suitable biochemical factors [882]. All previously mentioned clearly indicates that for the purposes of tissue engineering, calcium orthophosphates play only an auxiliary role; namely, they act as a suitable material to manufacture an appropriate 3D template, substrate or scaffold to be colonized by living cells before the successive implantation. However, the scaffolds themselves might be prepared from not only pure calcium orthophosphates but also organic-inorganic composites [883,884,885,886,887,888,889]. The *in vitro* evaluation of potential calcium orthophosphate scaffolds for tissue engineering has been described elsewhere [890], while the data on mechanical properties and porosity of calcium orthophosphates for use in tissue engineering are also available [891,892]. The effect of a HA-based biomaterial on gene expression in osteoblast-like cells was reported [893]. The influence of adsorbed serum proteins, RGD and proteoglycan-binding peptides on the adhesion of mesenchymal stem cells to HA was studied [894]. To conclude this part, the excellent biocompatibility of calcium orthophosphates, their possible osteoinductivity [745] and a high affinity for proteins and cells makes them very functional for hard tissue regeneration [895,896,897].

## 9. Conclusions and Outlook

By the end of the 20-th century, it became clear that calcium orthophosphate biomaterials and bioceramics by themselves could not provide a complete response to the clinical needs for artificial implants. Biomaterials with more demanding properties were required. Namely, in 1998, Hench published a forecast for the future of biomaterials development [898], where he noted that available that time bioactive materials (calcium orthophosphates, bioactive glasses and glass ceramics) had already improved prostheses lifetime but, unfortunately, any type of prosthesis had mechanical limitations. As the solution, he proposed that biomaterial researchers would need to focus on tissue regeneration instead of tissue replacement. A working hypothesis was announced: “Long-term survivability of prosthesis will be increased by the use of biomaterials that enhance the regeneration of natural tissues” [898]. One path to follow is the regeneration of bone using calcium orthophosphate scaffolds that mimic the structure of biological apatite, bond to bone and in some cases activate the genes within bone cells to stimulate new bone growth [667,823,881]. Thus, 10 years ago Hench predicted a rapid development of tissue engineering field, where calcium orthophosphates play an auxiliary role. The history has shown that tissue engineering, indeed, is a very rapidly developed field of science and research [899].

However, what can be said about calcium orthophosphates themselves? The major questions on chemistry, crystallization, ion-substitution, crystallography, thermodynamics and phase relationships for the chemically pure calcium orthophosphates have been answered in the 20-th century. Some topics for DCPD and CDHA have been additionally investigated in the field of calcium orthophosphate cements [686]. Conversely, calcium orthophosphates of biological origin, including the control of their morphology and interaction of calcium orthophosphate bioceramics with various bio- and organic compounds are not well investigated yet. The same is valid for nanocrystalline samples of calcium orthophosphates. Small amounts of bone-like apatite might be easily prepared by crystallization from SBF and rSBF but what can be said about larger quantities? A standard way of the concentration increasing causes chemical changes in the precipitates [797]. After a necessary technology is developed, one will have to think on scaffold preparation from this material, keeping in mind that any thermal treatment would destroy this material. A spark plasma sintering approach based on the use of pulsed current and enabling very fast heating and cooling rates seemed to be a first hint to achieve this goal [900]. However, a rapid development of calcium orthophosphate cements [686], which can be easily doped by the necessary chemical elements, seems to be a better solution of this problem. Furthermore, the existence of oxyapatite [68] remains to be questionable, as well as the bioactivity mechanism of calcium orthophosphates requires improving.

To date, although calcium orthophosphate biomaterials and bioceramics have been extensively studied for over 50 years, their ability to trigger bone formation is still incomparable with other biomaterials. Naturally, the biomaterials’ field is shifting towards biologically active systems in order to improve their performance and to expand their use [901]. Due to this case, tissue engineering is the strongest direction of current research, which, in the case of calcium orthophosphates, means fabrication of proper substrates and/or scaffolds to carry cells, hormones and biochemical factors to be further used in surgery and medicine. Presumably, a synthesis of various types of calcium orthophosphate-based biocomposites and hybrid biomaterials [743] occupies the second important place. For example, even composites with carbon nanotubes already exist [902,903,904]! The third important place is occupied by investigations devoted to the synthesis and characterization of various nanoparticles and nanocrystals of calcium orthophosphates [905,906], as well as by synthesis of calcium orthophosphates with controlled particle geometry [355]. In general, the geometry of crystal phases can be varied by controlling the precipitation conditions, such as temperature, solution pH, concentration of the reagents, hydrodynamics, presence of various admixtures, inhibitors or promoters, ultrasonication, *etc*. All these approaches might be useful in preparation of calcium orthophosphate fibers, whiskers, hollow microspheres, *etc*. In addition, a great attention is paid to manufacturing of calcium orthophosphate cements [686] and multiphase [907] mixtures mimicking as closely as possible the mineral component of biological apatite. A work along the ecological ways of synthesis of calcium orthophosphates might be of a great importance as well [909]. A deeper study of the fascinating growth rate of deer antlers and the ability of some animals, such as newts, to regenerate amputated limbs might provide new and unexpected approaches to the bone-healing concept, as well as this will be important for further development of both biomimetics and biomineralization fields. Unfortunately, no currently available grafting biomaterials can substitute the bones’ mechanical function, illustrating yet unmet medical need that would entirely substitute and regenerate a damaged tissue or organ. In a close future, the foreseeable application of calcium orthophosphates will be as a component of the third generation biomaterials [881,898], where they will support cells and/or other biologically active substances (peptides, growth factors, hormones, drugs, *etc*.) to guide regeneration of hard tissues [631,842,910,911,912,913,914,915]. To finalize this comprehensive review, one should note that, in spite of a long history of the calcium orthophosphate research and many important discoveries, still many gaps remain in our knowledge to be investigated in future.
